# Biomimetic hydrogel for the construction of patient-derived bladder cancer organoids with aggressive growth

**DOI:** 10.1016/j.isci.2026.114786

**Published:** 2026-01-27

**Authors:** Jin Zhang, Jiaxin Wang, Xiaofeng Hu, Wei Jia, Ziyuan Zhou, Gaohaer Kadeerhan, Wenmin Guo, Jun Tian, Hong Guo, Ling Guo, Dongwen Wang

**Affiliations:** 1 National Cancer Center/National Clinical Research Center for Cancer/Cancer Hospital & Shenzhen Hospital, Chinese Academy of Medical Sciences and Peking Union Medical College Shenzhen, Guangdong 518116, P.R. China; 2 Shanxi Medical University, Taiyuan 030001, China; 3 Department of the First Clinical Medical College, Shanxi Medical University, Taiyuan 030001, China; 4 Department of Urology, First Hospital of Shanxi Medical University, Taiyuan, Shanxi 030001, China

**Keywords:** Biological sciences, Bioengineering, Biomimetics, Stem cells research, Cancer, Biomaterials

## Abstract

In cancer, the extracellular matrix (ECM) reorganization, notably collagen, forming dense, thickened, and orderly structures, affects tumor traits. Bladder cancer organoids (BCOs) mimic tumor properties for personalized medicine. However, current organoid scaffolds lack tumor-such as collagen, crucial for cell growth and migration. Previous efforts to incorporate mesoscale collagen fibers extracted directly from tumors into scaffolds were limited by the size of the tumor tissue and the efficiency of extraction. In this study, we used cellulose microfibers (MCFs) to mimic *in vivo* mesoscale collagen’s role, enhancing BCO viability, invasiveness, and migration, aligning with tumor growth patterns. These organoids preserved tumor architecture and mutations, showing drug sensitivities similar to those of parental tissue-derived cells and correlating with patient outcomes. This suggests that such organoids can serve as preclinical models to inform therapeutic strategies.

## Highlights

• ECM reorganization, especially collagen, influences cancer traits and tumor behavior
• Bladder cancer organoids mimic tumor properties for personalized therapeutic models
• Cellulose microfibers enhance organoid viability, invasiveness, and migration
• Organoids preserve tumor architecture and mutations, reflecting drug sensitivities

## Graphical abstract

**Figure:**
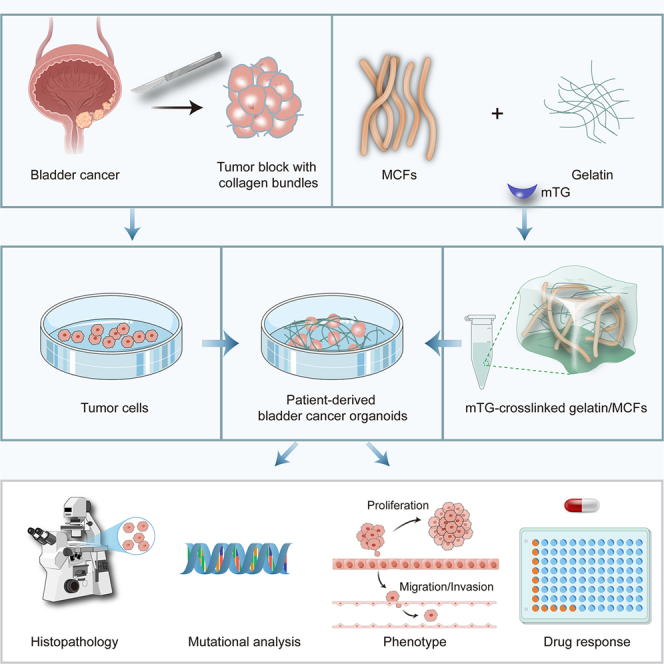

## Introduction

Globally, bladder cancer ranks as the second most prevalent malignancy within the urogenital system, surpassed only by prostate cancer.^1^ It can be categorized into non-muscle-invasive (NMIBC) and muscle-invasive (MIBC), with NMIBC being the predominant form at 80% of diagnoses. The prevalent treatment for NMIBC is transurethral resection, followed by intravesical chemotherapy to enhance the therapeutic outcome.^2^^,^^3^ Nevertheless, the management of tumors in clinical settings is less than ideal, with recurrence rates estimated between 15% and 61% within the first year and escalating to 31%–78% within a five-year period.^4^ While MIBC is relatively rare, its treatment necessitates aggressive measures such as radical cystectomy or chemotherapy, which can profoundly affect the patient’s well-being.^5^ Additionally, the propensity for metastasis frequently emerges as the primary determinant of mortality in such cases.^6^ Hence, establishing a reliable model is essential for delving into the root causes of bladder cancer’s recurrence, advancement, and dissemination, thereby offering novel approaches for its detection and management.

Thanks to advances in biomaterials and tissue engineering, we are hopeful about constructing *in vitro* models that accurately simulate the tumoral and intertumoral heterogeneity and complex biological regulatory mechanisms exhibited by tumor cells in the human body.^7^^,^^8^ Tumor organoids, emulating the natural cellular and tissue growth, provide a detailed understanding of disease processes that exceeds that of conventional *in vitro* cultures and experimental animal studies, and are strategically utilized in disease modeling, biobanking, mechanism exploration, and drug screening to foster the advancement of personalized clinical medicine.^9^^,^^10^^,^^11^^,^^12^^,^^13^ Organoids have been constructed for cancers such as lung,^14^^,^^15^^,^^16^ kidney,^17^^,^^18^ bladder,^19^^,^^20^ breast,^21^^,^^22^ colon,^23^^,^^24^ and prostate,^25^^,^^26^ serving as a conduit between the realms of basic research and clinical implementation, and facilitating the formulation of personalized treatment strategies.

Physical properties, including solid stress, interstitial fluid pressure, matrix stiffness, and architecture, are pivotal in cancer’s genesis, progression, immune evasion, and therapeutic response.^27^^,^^28^ Throughout the progression of neoplasms, extracellular matrix (ECM) experiences substantial reconfiguration. Particularly, collagen fibers form a dense, thickened, and orderly arrangement, a characteristic observed in various tumor types rather than being confined to a single subtype.^29^^,^^30^ This has been documented in primary tumors of the human bladder,^31^^,^^32^^,^^33^^,^^34^^,^^35^ brain,^36^ prostate,^37^ and breast.^38^ For example, in brain tissue samples from patients with glioblastoma multiforme, collagen fiber bundles were found to have a width greater than 2 μm.^36^ Reconstituted collagen gels or matrix gel, which are widely used as standard models for studying tumor cell-ECM interactions and tumor organoid culture, exhibit a reticular, isotropic nanofiber structure with an average diameter of approximately 900 nm.^39^^,^^40^ However, they fail to recapitulate the dense, aligned, and thick collagen bundles observed *in vivo*.^40^^,^^41^^,^^42^

Research on medium-scale collagen bundles has primarily focused on the development of biomimetic scaffolds that recapitulate the structural and biochemical features of the *in vivo* tumor stroma for use *in vitro* tumor models. However, conventional approaches present several limitations. Electrospinning enables the production of aligned microscale polymeric fibers but relies on synthetic materials that lack the native ligand-binding domains of collagen and requires specialized equipment.^40^^,^^43^ Meanwhile, 2.5D micropatterned surfaces offer tunable alignment and dimension control but fail to reproduce physiologically relevant three-dimensional (3D) microenvironments.^44^^,^^45^ In contrast, recent advancements in type I collagen-based microscale systems have demonstrated significant progress. One approach leverages controlled temperature, diluent composition, and ionic strength to fabricate type I collagen bundles (4–9 μm in diameter), which can be directionally aligned using microfluidic platforms to induce contact guidance and directional migration of cancer cells, or embedded within agarose hydrogels to support cancer cell outgrowth.^40^ Another method modulates collagen fibrillogenesis and gelation through mechanical agitation, generating scaffolds featuring large-scale, thickened, wavy collagen regions interconnected by loose fibrillar networks—architectures that closely resemble both tumor stromal and skin scar ECMs.^46^ These structures promote tumor cell dissemination while inducing changes in cellular morphology and migratory behavior. Despite these advances, current collagen sources—typically derived from rat tail tendons or bovine hide, commonly used to simulate native collagen bundles—are constrained by high procurement costs and substantial batch-to-batch variability due to inherent biological differences among donors.^40^

To address this issue, our research group previously used fragments from tumor tissues to extract mesoscale collagen (MC) fibers, with a mean length of 90 ± 27 μm and a diameter of 5 ± 1.5 μm, to effectively replicate the tissue structure. However, this method, although effective, is limited by the size of the collected tumor tissue and the digestion capacity, restricting the generation of collagen bundles.^47^ The utilization of cellulose-based biopolymers is proving to be an exceptionally promising method for fabricating sustainable composite materials. Cellulose microfibers (MCFs) and cellulose nanocrystals (CNCs) are the two primary forms of cellulose. CNCs have been successfully applied in culturing breast cancer organoids,^48^ while MCFs, known for their distinctive attributes such as a high elastic modulus, increased aspect ratio, impressive tensile strength, substantial specific surface area, reduced density, biodegradability, and low energy demand, have been successfully integrated into composite material applications.^49^

In this study, we introduced MCFs to simulate the tissue structure of the ECM, overcoming the limitations of tumor tissue size and extraction efficiency. Initially, MCFs exhibit a form similar to the MC previously extracted from the organization.^47^ Our results indicate that the addition of MCFs in bladder cancer organoids (BCOs) culture significantly enhances cell viability. Moreover, they maintain other tumor characteristics relevant to the recurrence of bladder cancer, such as migration and invasion capabilities.^50^ These organoids retain the tissue structure and mutation spectrum of the parental tumor. In drug response tests, organoids closely replicate those in tumor tissues, highlighting the potential of this model as a platform for drug testing. Using clinically relevant intravesical drugs (such as pirarubicin and mitomycin C), we found that the post-treatment adhesion capacity of organoids correlates with patient follow-up results, preliminarily exploring the correlation between organoid construction and clinical outcomes. Furthermore, organoids cultured with MCFs demonstrated consistent cell viability, cellular morphology, and drug response across different passage numbers. These findings emphasize the fidelity of our organoid model in simulating the growth characteristics of tumors *in vivo* and provide a superior simulation method for ECM remodeling in organoid culture, as well as a feasible approach for long-term development.

## Results

### Synthesis of enzymatically cross-linked gelatin/cellulose microfiber-composite hydrogels

**Figure 1:**
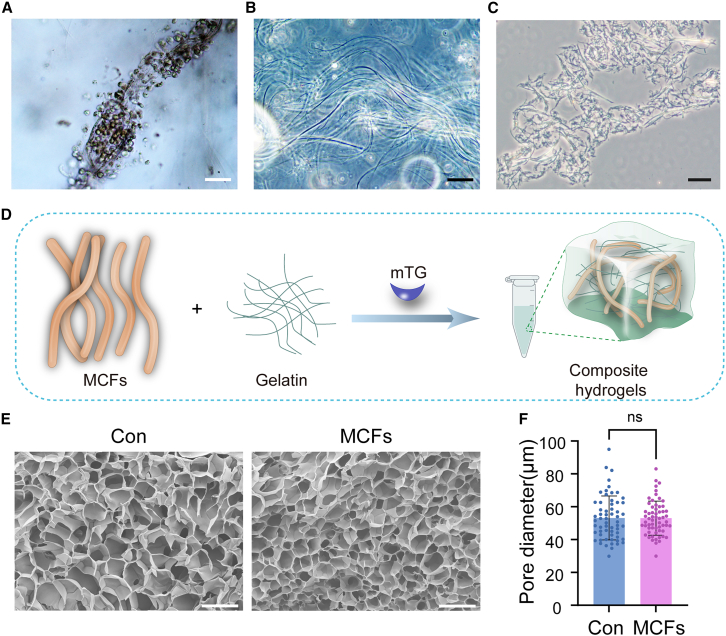
The characterization of composite hydrogels consisting of microbial transglutaminase (mTG) crosslinked gelatin/cellulose microfibers (MCFs) (A) Bright-field microscopy images depict the clustering and adhesion of bladder cancer cells within collagen bundles. Scale bars, 200 μm. (B) Bright-field images show the characteristics of collagen bundles in undigested tissue debris after the removal of bladder cancer cells. Scale bars, 100 μm. (C) Bright-field images illustrate the characteristics of MCFs, serving as a substitute for collagen bundles. Scale bars, 100 μm. (D) Summary diagram of the preparation of mTG-crosslinked gelatin/MCFs. (E–F) SEM images of MCFs and the control hydrogel. Pore diameter was calculated by ImageJ. Scale bars, 100 μm. The data were reported as the mean ± SD. Data in (F), *n* = 60, ns: not significant, unpaired two-tailed Student’s *t* test.

Solid tumors have an upregulation in stromal collagen expression, resulting in the formation of tightly packed, thickened, straightened, and well-aligned bundles.^40^ The bladder cancer tissues underwent mechanical disruption and enzymatic digestion to separate single bladder cancer cells from large, undigested tissue debris. Upon microscopic examination, the undigested tissue debris showed that tumor cells aggregate within collagen fibers (Figure 1A). After the removal of tumor cells, the retained undigested tissue debris was found to contain an abundance of collagen strands tightly bundled together (Figure 1B). To mimic these mesoscale collagen bundles *in vivo*, we employ MCFs as a substitute for collagen to promote the formation of BCOs. The MCFs we employed had lengths ranging from 100 μm to 200 μm and an average diameter of approximately 4 μm–8 μm, which were comparable in thickness to the collagen bundles we previously obtained from tumor tissue-derived ECM debris (Figure 1C). Enzymatically cross-linked gelatin was utilized as a scaffold (Figure 1D).

The structural characteristics of the MCFs hydrogel and the control hydrogel (gelatin crosslinked with mTG, excluding MCFs) were analyzed using scanning electron microscopy (SEM). No discernible MCFs were detected within the pore walls, suggesting a strong interfacial compatibility between the MCFs and the gelatin matrix (Figure 1E). This finding aligns with previous studies that have reported similar observations.^47^^,^^51^ Furthermore, similar to our previous study, both the MCFs hydrogel and the control hydrogels demonstrated a unique and intricate porous structure with uniformly smooth pore walls. A comparison of pore sizes (Figure 1F) showed similarity between the two, suggesting that the inclusion of MCFs had no impact on the scaffold’s structural properties.^51^

### Cellulose microfiber-combined hydrogels enhanced the growth of bladder cancer organoids

**Figure 2:**
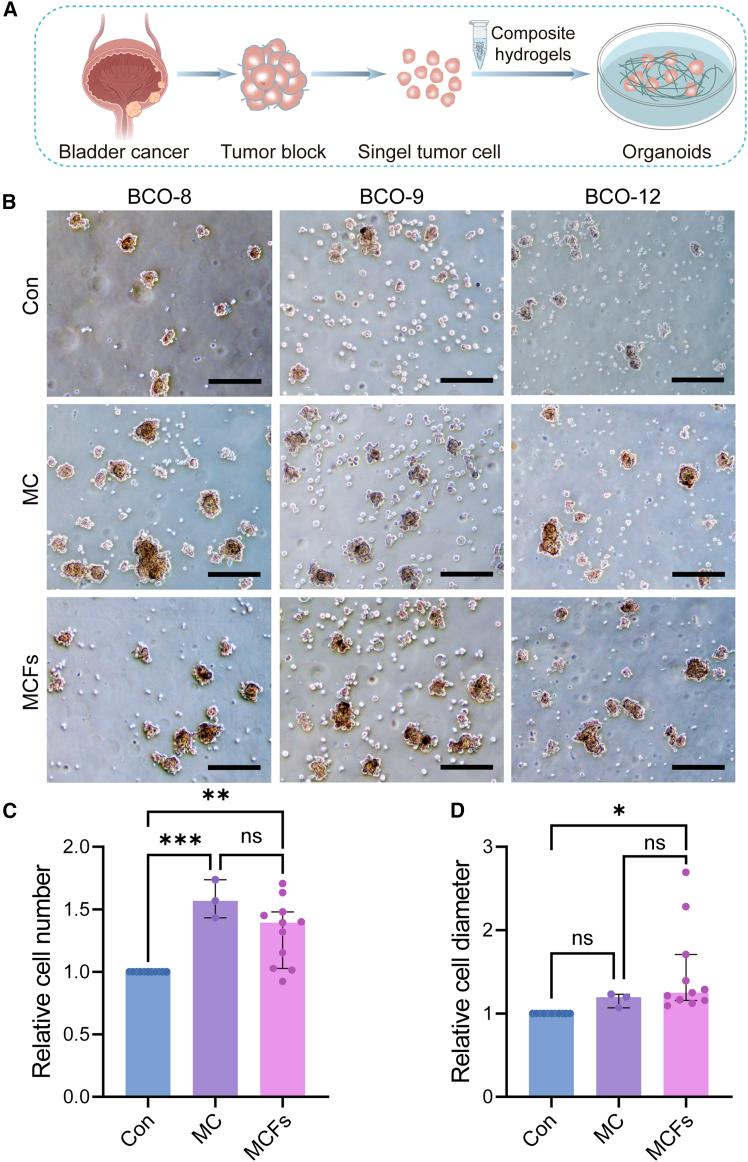
Enhanced viability of bladder cancer organoids (BCOs) with MCF-combined hydrogels (A) Schematic illustration of BCOs fabrication utilizes MCF-combined hydrogels. (B–D) Representative bright-field images and corresponding measurements of relative cell numbers and diameters of BCOs cultured for 10–14 days under control, mesoscale collagen fibers (MC), and MCF conditions. The relative cell number and relative cell diameter in (C–D) were measured by randomly capturing bright-field images, including all cells within the captured areas. Scale bars, 200 μm. Data in (C–D) were displayed as median with interquartile range (IQR). Data in (C–D), biological replicates: Con *n* = 11; MC *n* = 3; MCFs *n* = 11, ∗*p* < 0.05, ∗∗*p* < 0.01, ∗∗∗*p* < 0.001, Kruskal-Wallis test with Dunn’s post hoc test.

We investigated the growth capacity of organoids within the MCFs hydrogel, as shown in the schematic representation (Figure 2A). A total of 17 bladder tumor tissue samples were collected, obtained from transurethral resection, laser surgery, partial cystectomy, and radical cystectomy. Using organoid culture medium, the composition and details of which are provided in Table S1, we successfully established organoids from 14 of these bladder cancer cases. The remaining three cases did not form organoids, possibly due to inadequate tumor tissue size or low cell viability after transurethral resection or laser surgery. Detailed clinical information for the patients was provided in Table S2, and the passage numbers for the BCOs were presented in Table S3.

To validate the consistency of MCFs as an alternative to MC that we previously used to construct organoids for promoting BCOs growth, organoids were cultured under the indicated conditions, as shown in Figure 2B and Figure S1. Owing to limited MC supply, Figure 2B displays results from three sample sets assessed under Con, MC, and MCFs. In addition, eight independent samples (Figure S1) were examined under two conditions (Con and MCFs), for a total of 11 independent samples. The results demonstrated that both MCFs and MC significantly enhanced organoid proliferation, as evidenced by increased viability and larger diameters (Figures 2C and 2D). Notably, no significant differences were observed between the two conditions, indicating that MCFs and MC exert comparable effects on organoid growth (Figures 2C and 2D).

Additionally, throughout the entire cultivation process, the MCFs consistently exhibited a well-defined fibrous structure, ensuring the stability of the MCFs hydrogel. BCOs cultivated in MCFs hydrogel demonstrated an irregular growth pattern, along with significantly higher cell counts and increased cell diameters (Figures 2B–2D and S1).

### Bladder cancer organoids cultured in cellulose microfiber-combined hydrogels recapitulated the histological features of parental tumors

**Figure 3:**
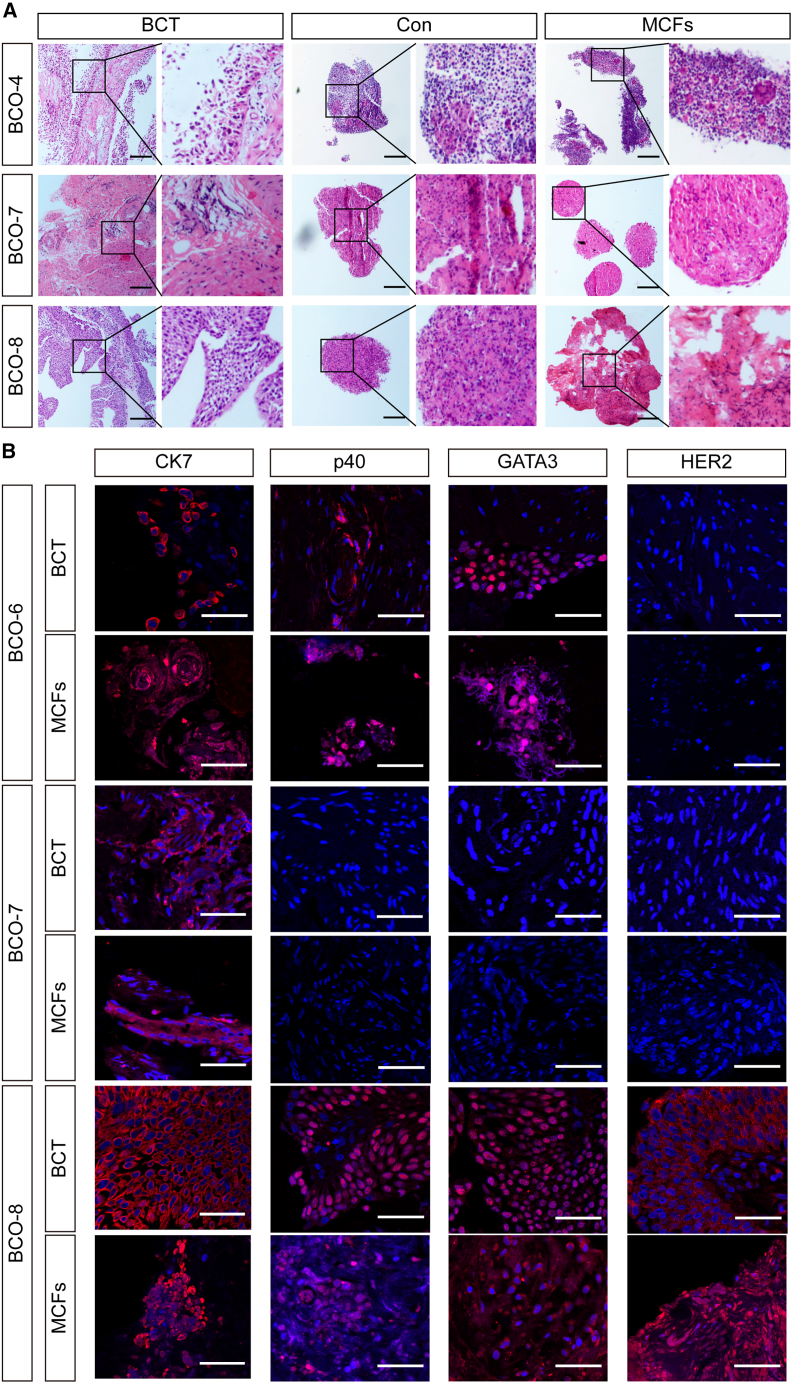
Culture of BCOs in MCFs-enriched hydrogels recapitulates the histopathological architecture of parental tumors (A) Representative H&E staining images of BCOs cultured in MCFs and control conditions, as well as corresponding parental bladder cancer tissues (BCTs). Scale bars, 200 μm. (B) Representative immunofluorescence images of CK7, p40, GATA3, and HER2 in BCOs and corresponding BCTs with nuclear staining by DAPI (blue). Scale bars, 50 μm.

Hematoxylin and eosin (H&E) staining was conducted to compare the morphological characteristics of parental bladder cancer tissue (BCT) with those of organoids cultured under control (Con) and MCFs conditions (Figure 3A). The organoids and their corresponding source tissues exhibited broadly similar architectural features. Specifically, BCT displayed complex papillary structures characterized by well-developed fibrovascular cores, high cellular density, and marked nuclear atypia. Likewise, both Con-derived and MCFs-derived organoids formed densely packed spheroidal structures, occasionally containing gland-like formations and surrounded by fibrous-like extracellular material adjacent to tightly clustered tumor cells; however, this stroma-like appearance was not indicative of true stromal tissue, particularly in MCFs-derived organoids. The observed fibrous morphology is likely attributable to the deposition of extracellular matrix components within the MCFs-based hydrogel, which partially recapitulates stromal architecture. Notably, due to the absence of immune cells, vascular structures, and the inherent heterogeneity of the original tumor tissue, neither Con- nor MCFs-derived organoids fully replicated the intricate tissue architecture, stromal organization, or inflammatory microenvironment present in native BCT.

Immunofluorescence staining was employed to assess the consistent expression patterns of tumor-associated markers in BCOs and original bladder cancer tissues, thereby validating the ability of the organoids to faithfully replicate the structure of the parent tumor. It was observed that positive staining for CK7, p40, and GATA3 was observed in both BCO-6 and its corresponding parental tissue. Positive staining for CK7 was also identified in both BCO-7 and its corresponding tissue. In BCO-8 and its parental tissue, we detected positive immunofluorescent signals for CK7, p40, GATA3, and HER2 (Figures 3B and S2). These results collectively highlight the organoids' ability to accurately replicate the histological features of bladder cancer.

Additionally, the consistent expression of Ki-67 and staining of F-actin in organoids and tumor tissues of BCO-8 and BCO-3 reflected cellular proliferation and morphological integrity in both the BCOs and their parental tissues (Figure S3). This demonstrates that the constructed organoids in MCF-combined hydrogels exhibit proliferative activity and maintain cellular integrity.

### Bladder cancer organoids cultured in cellulose microfiber-combined hydrogels preserved the mutational landscape of the original tumors

In our quest to verify the fidelity of genetic mutations from the progenitor tumors within the hydrogel, which contained MCFs, a detailed exomic sequencing analysis was undertaken. This involved a comparative study of six sets of BCT samples, and their derived BCOs cultured in MCFs-infused hydrogel, aiming to discern the preservation of genetic traits. To identify somatic mutations, variants were filtered, and polymorphisms common to both organoid lines and the corresponding parental tumors were excluded by comparing them with matched normal blood samples from the patients.

**Figure 4:**
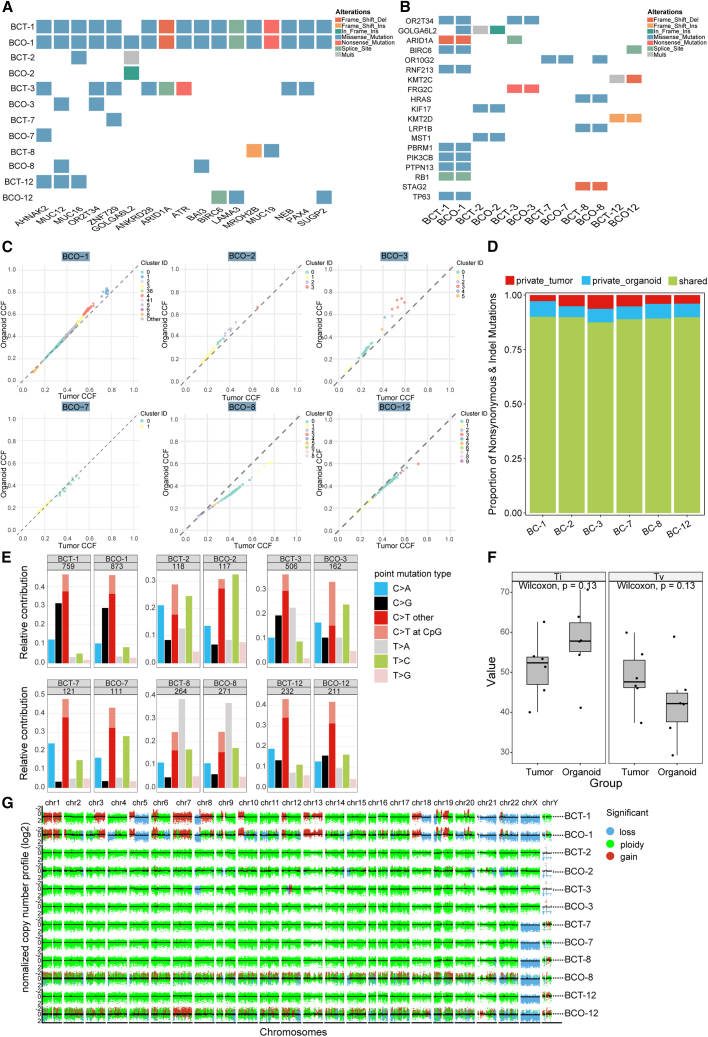
BCOs cultured in MCFs-infused hydrogels retained the genetic characteristics of the original BCTs (A) Heatmap analysis of the top 17 genes in BCOs cultured in MCFs-infused hydrogel and BCTs. (B) The heatmap illustrates somatic mutations in cancer-related genes, as detected in the TCGA study and known to be associated with bladder cancer, in BCOs cultured in MCFs-infused hydrogel and corresponding tissue samples. (C) Scatterplot shows the differences in cancer cell fraction (CCF) between the genetic variations that occurred in BCOs cultured in MCFs-infused hydrogel and parental tissue samples. (D) Comparison of nonsynonymous mutations and indels between BCOs and parental tissue samples. (E) The proportions of exonic variants in BCOs cultured in MCFs infused hydrogel compared to their parental tumors. The legend details the seven types of base substitutions, and the numbers at the top indicate the mutation counts. (F) Percentages of transitions (Ti) and transversions (Tv) for the two groups were shown. (G) Scatterplots illustrate the genome-wide CNVs of paired BCOs and BCTs.

Among the organoids analyzed, BCO-1 stood out with a significant alignment to its tissue of origin. In contrast, the remaining organoids and their corresponding tissues showed a diminished correlation in the mutation profiles of the top 17 genes, as illustrated in Figure 4A. This discrepancy may be attributed to the presence of mutations unique to the tissue, the inherent characteristics of the tissue, and the inherent limitations of the mutation detection methodologies. As shown in Figure 4B, the somatic mutations affecting cancer genes, based on TCGA data (referenced in Table S5) and known to be associated with bladder cancer,^52^^,^^53^^,^^54^^,^^55^^,^^56^^,^^57^^,^^58^ were remarkably consistent between the BCOs and their parental tissues, reflecting diverse mutational spectra. Notably, cancer-related genes such as ARID1A,^59^ BIRC6,^60^ KMT2C,^61^ HRAS,^62^ KMT2D,^63^ LRP1B,^64^ PBRM1,^65^ STAG2,^66^ RB1,^67^ and TP63^68^ were conserved between the BCOs and their corresponding BCTs, with BCO-1 and its originating tumor exhibiting a high degree of similarity. Further, the cancer cell fraction (CCF) analysis (Figure 4C) demonstrated a high degree of consistency between the organoids and their corresponding parental tumor tissues, indicating that the organoids largely preserve the clonal architecture of the original tumor tissue. Additionally, the analysis of nonsynonymous mutations and indels (Figure 4D) showed that the organoids maintain a similar mutational profile to the parental tumor tissue. These findings suggest that the organoid model faithfully replicates the genetic alterations of the original tumor, further supporting its utility as a robust *in vitro* model for studying tumor-associated mutations and clonal evolution.

To delve deeper into the genetic conservation between the BCOs and their BCTs, we conducted an in-depth analysis of somatic mutation patterns within the tumor samples and the organoids cultured in the MCFs hydrogel. In terms of base substitutions, the predominant types observed in both the BCTs and the BCOs within the MCFs-infused hydrogel were transitions of C to T or T to C (Ti) and transversions of C to A or C to G (Tv). Conversely, transversions of T to G were the least frequent mutation type, as represented in Figure 4E. This is consistent with the mutational spectrum reported in bladder cancer.^69^^,^^70^ Additionally, the fundamental patterns of somatic mutations were well preserved (Figure 4F). As shown in Figure 4G, copy number variation (CNV) analysis revealed similar patterns of DNA copy number gains and losses between the bladder cancer organoid lines and their corresponding tumors, except for BCO-8 and BCO-12. These discrepancies may be attributed to tissue sampling variations from other organoid-derived sources.^71^ Notably, BCO-1 exhibited a high degree of similarity, consistent with the results in Figures 4A and 4B.

### Cellulose microfiber-combined hydrogels facilitated the migration and invasion capabilities of bladder cancer organoids

**Figure 5:**
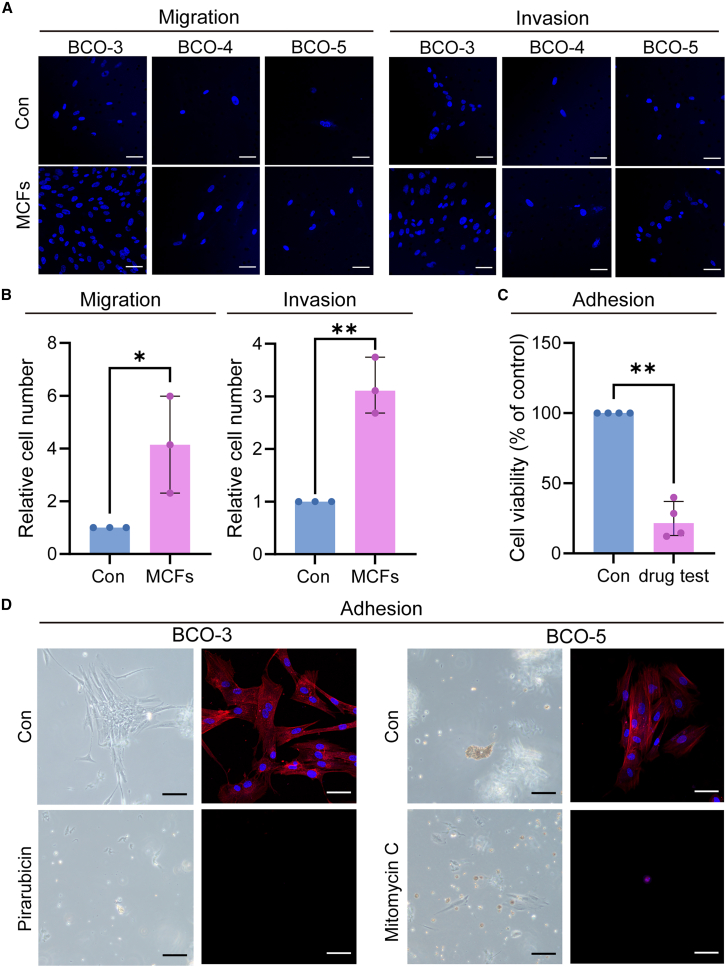
Enhanced migration, invasion, and clinical efficacy of BCOs in MCFs-infused mTG-crosslinked gelatin: migration, invasion, and adhesion assays with drug treatment under MCFs conditions (A and B) Representative immunofluorescence images of BCOs under control and MCFs conditions, with relative cell numbers indicating migration and invasion capacities, respectively. Nuclei are stained with DAPI (blue). Scale bars, 50 μm. Relative cell numbers in (B) were measured by randomly capturing immunofluorescence images and counting all cells within the captured areas. (C) Viability of four BCO lines established under MCFs conditions exposed to mitomycin C or pirarubicin, with control as the blank counterpart, was assessed following a cell adhesion assay. Adherent cell viability in (C) was assessed using the CellTiter-Lumi Luminescent Cell Viability Assay kit. (D) Representative bright-field and immunofluorescence results after adhesion assay. In the bright-field images, the black scale bar represents 200 μm, while in the immunofluorescence images, the white scale bar represents 50 μm. Nuclear staining by DAPI (blue) and F-actin staining (red). Data in (B–C) are displayed as median with IQR. Data in (B), biological replicates: *n* = 3; data in (C), biological replicates: *n* = 4. ∗*p* < 0.05, ∗∗*p* < 0.01, unpaired two-tailed Student’s *t* test.

The high recurrence rate of bladder cancer, particularly NMIBC, is attributed to the tumor’s migratory and invasive capabilities.^4^^,^^50^ To further validate the efficacy of our organoid model, we examined the tumor migratory and invasive phenotypes of organoids under conditions with and without MCFs. We performed functional assays on three distinct BCO lines, cultivated in MCFs-infused mTG-crosslinked gelatin hydrogel and in the control hydrogel. Notably, BCOs in the MCFs-enriched hydrogel exhibited significantly enhanced migratory capabilities compared to the group without MCFs (Figures 5A and 5B). Additionally, the invasive capacity of the organoids was markedly increased in the presence of MCFs (Figures 5A and 5B).

### Bladder cancer organoids in cellulose microfiber-combined hydrogels evaluated the efficacy of postoperative bladder drug instillation

The postoperative intravesical drug instillation is commonly performed in clinical practice for patients with bladder cancer to effectively prevent tumor recurrence and reduce the risk of progression. To assess the impact of postoperative intravesical drug instillation, we selected four organoid lines (BCO-3, BCO-5, BCO-9, and BCO-11) derived from individuals who underwent postoperative intravesical instillation with either pirarubicin or mitomycin C. The primary follow-up indicator was cystoscopic examination conducted three months post-surgery, as referenced in the clinical guidelines; the absence of new tumor growth or lesions on the bladder wall indicated effective treatment. After drug treatment, there was a significant reduction in the viability of the four organoid cell lines (Figure 5C) cultured in MCFs hydrogel. Furthermore, BCO-3 and BCO-5 demonstrated a marked reduction in both the number of organoids with adhesive capacity and the extent of fragmentation following drug treatment (Figure 5D). This finding is consistent with the cystoscopic follow-up results of the patients (Table S4), suggesting BCOs in MCFs hydrogel serve as a valuable platform for predicting the efficacy of postoperative intravesical drug instillation.

### Bladder cancer organoids cultured in cellulose microfiber-combined hydrogels maintained clinically relevant drug tests

**Figure 6:**
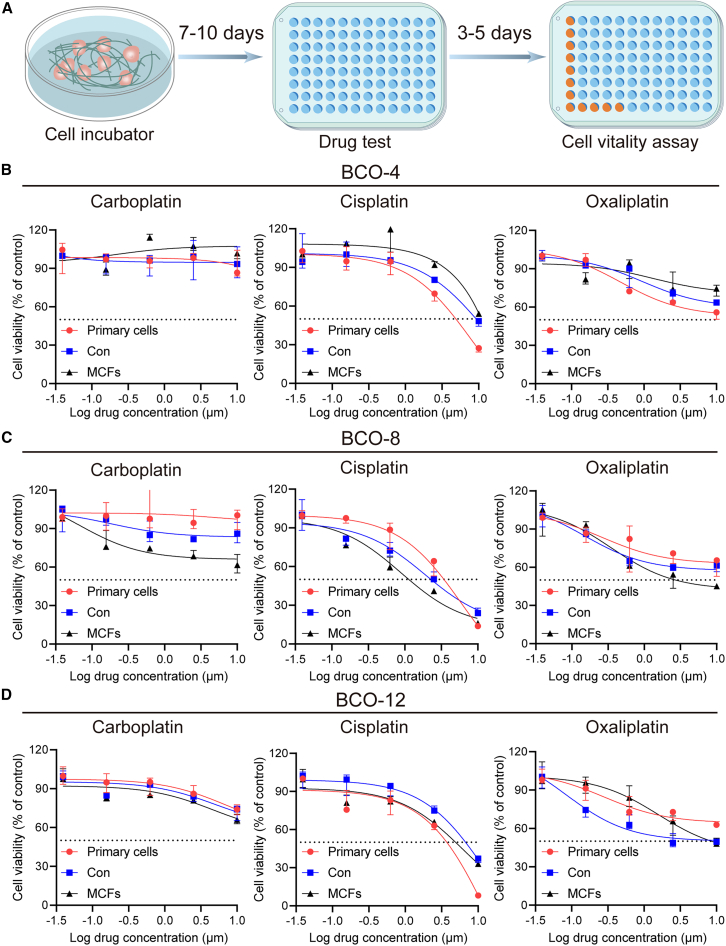
Drug experiments on BCOs under control and MCFs conditions and in corresponding tissue-derived cells (A) Schematic overview of the drug experiment procedure for BCOs. (B–D) Three BCO lines were treated with cisplatin, carboplatin, and oxaliplatin under both control and MCFs conditions, as well as in the corresponding tissue-derived cells. Cell viability in (B–D) was assessed using the CellTiter-Lumi Luminescent Cell Viability Assay kit, and concentrations were log-transformed. Data of (B–D) were displayed as median with IQR. Data in (B–D), *n* = 3.

We subsequently evaluated the sensitivity of three organoid lines (BCO-4, BCO-8, BCO-12) to three platinum-based drugs: cisplatin, carboplatin, and oxaliplatin. The organoids were cultured under both MCFs and control conditions. To further validate whether the drug responses of organoids closely mimic patient responses, we conducted drug sensitivity assays on tumor cells directly obtained from patients. Following a 7-10-day culture period, the organoids were exposed to the drugs for 3–5 days, after which cell viability was measured under identical conditions with replicates (Figure 6A). Drug sensitivity was assessed based on relative viability. The drug response of BCOs cultured in MCFs exhibited similar trends to those observed in the control group and corresponding tissue-derived cells (Figures 6B–6D).

In addition, the organoids from BCO-4, BCO-8, and BCO-12 under both culture conditions, as well as the parental tissue-derived cells, exhibited resistance to both carboplatin and oxaliplatin. For BCO-4, primary cells showed slight sensitivity to cisplatin, while organoids cultured in both MCFs and control conditions exhibited resistance. In contrast, BCO-8 and BCO-12 showed sensitivity to cisplatin under both conditions, similar to that observed in parental tissue-derived cells. These findings suggest that the drug responses observed in the constructed organoids closely replicate those in tumor tissues.

### Cellulose microfiber-combined hydrogels enable the establishment of the long-term culture of bladder cancer organoids

**Figure 7:**
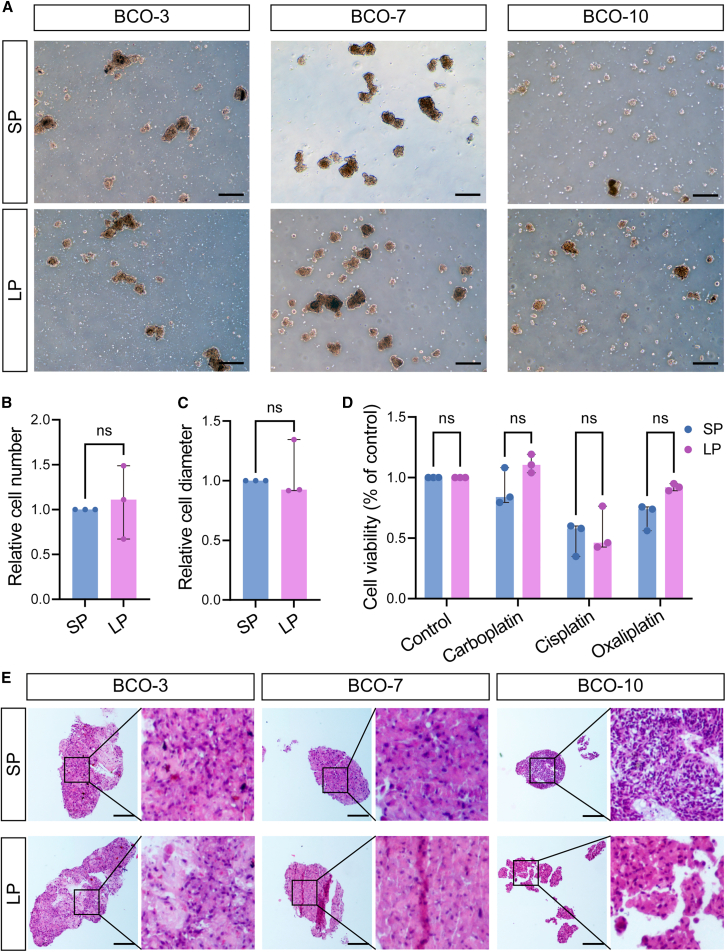
Growth characterization and drug testing of long-term organoids in MCF-combined hydrogels (A–C) Representative bright-field micrographs of BCOs cultured for an extended period (P7/8) and a shorter duration (P2) in MCFs, with their numbers and diameters quantified. Scale bars, 200 μm. The relative cell numbers and relative cell diameters in (B–C) were measured by randomly capturing bright-field microscopy images, including all cells within the captured areas. (D) Viability of long-term cultured BCOs at P7/8 and early-passage BCOs at P2 in MCFs was assessed using the CellTiter-Lumi Luminescent Cell Viability Assay kit after exposure to cisplatin, carboplatin, and oxaliplatin, with relative cell survival measured. (E) H&E staining of organoids cultured under MCFs conditions for both long-term and short-term periods. Scale bars, 200 μm. Data in (B–D) are plotted as median with interquartile range. Data in (B–D), biological replicates: *n* = 3, ns: not significant, unpaired two-tailed Student’s *t* test. SP refers to short-term passage, and LP refers to long-term passage.

Among the 14 bladder cancer cases, three BCOs were successfully cultured under long-term conditions using MCF-combined gel. These organoids exhibited sustained cell growth potential, with consistent split ratios maintained even at later passages, up to passages 7/8 (Figures 7A–7C). Notably, they could be propagated for over three months with a minimum splitting ratio of 1:2, without a significant decline in cell proliferation capacity (Figures 7A–7C). In addition, these organoids also exhibited high viability following cryopreservation for more than three months and were able to expand upon thawing. The long-term established BCOs in MCFs maintained drug sensitivity profiles comparable to those of their short-term counterparts (Figure 7D). This highlights the potential for utilizing long-term cultured BCOs as a model for personalized treatment strategies in bladder cancer. Furthermore, H&E morphology analysis revealed that organoids cultured in MCFs retained consistent morphological features across both short-term and long-term culture periods (Figure 7E).

## Discussion

In this study, we successfully established organoids from 14 BCOs derived from 17 patients with cancer using MCF-enriched hydrogel. In this model, BCOs demonstrated enhanced growth, migration, and invasion capabilities while maintaining their architectural and genetic integrity. Notably, BCOs embedded in MCF-enriched hydrogel retained acute drug sensitivity and served as a predictive tool for the efficacy of postoperative bladder drug instillation. The utilization of MCF-infused hydrogel for constructing organoids from bladder cancer tissues demonstrates the practicality and effectiveness, enhancing our grasp of tumor biology. This methodology simultaneously opens avenues for the identification of innovative treatments and the refinement of therapeutic strategies.

We investigated the impact of collagen architecture on tumor growth by using MCFs and microbial transglutaminase (mTG) to crosslink gelatin, forming a synthetic hydrogel. The utilization of mTG-crosslinked gelatin as a scaffold for patient-derived tumor organoids has been proven effective due to its favorable chemical and physical properties. MCFs in our organoid model have been crucial for replicating the complex collagen bundle structures characteristic of the tumor extracellular matrix. The patient-derived lung cancer organoids were successfully established in our previous investigation by extracting collagen bundles from tumor tissues.^47^ In this study, we utilized artificially synthesized MCFs to mimic *in vivo* collagen bundles and subsequently constructed patient-derived BCOs. This approach proves to be more efficient for large-scale production compared to methods involving the extraction of collagen bundles from tumor tissues, thus meeting the requirements for constructing patient-derived BCOs and establishing a biobank.

These collagen bundles, prevalent in the tumor microenvironment, provide crucial structural support and mechanical cues to resident cells, significantly influencing their migration and invasion.^72^^,^^73^^,^^74^ In our BCO models, the strategic use of MCFs has been pivotal. These fibrils, similar to those found in tumor tissues, are integral to the tumor extracellular matrix. BCOs in our study, supported by these mesoscale scaffolds, show enhanced growth activity and morphological irregularities that reflect the complexity of tumor cells in their natural microenvironment. Examination of malignant phenotypes revealed that MCFs significantly enhance the organoids' migratory and invasive capabilities, which are hallmarks of cancer progression.^75^ Notably, these organoids preserve their tissue morphology and genetic mutation profiles, consistently reflecting the original tumor’s heterogeneity.

Furthermore, the drug treatment assays with our organoids reveal nuanced therapeutic responses. Incorporating MCFs, organoids effectively replicate tumor tissues in drug sensitivity assays. The noteworthy aspect is that MCFs can more accurately reflect the drug responsiveness of clinical patients, potentially reversing the sensitivity pattern observed in traditionally constructed organoids. Applying clinically used anticancer drugs, such as pirarubicin and mitomycin C, to our MCFs-enriched organoids has produced drug response profiles closely correlated with patient outcomes after chemotherapy. Additionally, incorporating MCFs extends the culture period, supports sustained growth of BCOs, maintains cellular morphology, and preserves their drug sensitivity. This correlation supports the credibility of our findings and underscores the potential of our organoid system for personalized medicine. Our model captures the complexity of individual tumor biology and its dynamic interaction with therapeutic agents, paving the way for tailored treatment strategies that address bladder cancer’s heterogeneity.

### Limitations of the study

The current study’s shortcomings lie in the insufficient representation of the intricate nature of the Tumor Microenvironment (TME), particularly in relation to immune cells and vascular endothelial cells. These elements are essential for the complex cellular dialogues that unfold within the TME, influencing both intra-tumoral communications and the tumor’s interaction with its microenvironment. Furthermore, although the experiments conducted on the available samples demonstrated that the MCF-enriched hydrogels effectively recapitulate the tumor growth environment *in vivo*, the limitation in tissue availability prevented us from performing all experiments on all 14 patient samples. This constraint may impact the broader applicability and generalizability of our findings. Particularly, it should be noted that the characterization of luminal/basal subtypes, which are predictive of therapy outcomes and pose challenges for the clinical efficacy of organoids, was not included in this study.^19^^,^^20^ Moreover, the stratification of driver and passenger mutations was deferred. Future research will focus on integrating additional key cellular components into the tumor microenvironment model, optimizing culture efficiency, extending organoid cultures, investigating the mechanisms underlying the phenotypic shift, and exploring the stratification of driver and passenger mutations. These efforts aim to refine the model’s precision and to deepen the comprehensive understanding of the TME’s evolution. In addition, although organoid cultures were established using tumor tissues from both male and female patients with bladder cancer, the limited sample size and the lack of sex-stratified analyses prevented us from assessing the potential influence of sex on the experimental outcomes.

## Resource availability

### Lead contact

Requests for further information and resources should be directed to and will be fulfilled by the lead contact, Dongwen Wang (urology2007@126.com).

### Materials availability

This study did not generate new unique reagents.

### Data and code availability

• Raw WES data have been deposited at the NCBI Short Read Archive (SRA) as Database: PRJNA1162232 and are publicly available as of the date of publication. All other data reported in this work will be available from the lead contact upon request.
• This article does not report original code.
• Any additional information required to reanalyze the data reported in this article is available from the lead contact upon request.

## Acknowledgments

This research was funded by the Shenzhen Medical Research Fund (D2301001, B2402032), the 10.13039/501100001809National Natural Science Foundation of China (82273308, 82472934, 22107045); the Shenzhen Science and Technology Program (JCYJ20220530112817040, ZDSYS20220606101604009, GJHZ20220913142804008); the Shenzhen Key Medical Discipline Construction Fund (SZXK013); the 10.13039/501100012151Sanming Project of Medicine in Shenzhen (SZSM202111003); the National Cancer Center/National Clinical Research Center for Cancer/Cancer Hospital & Shenzhen Hospital, and the 10.13039/501100005150Chinese Academy of Medical Sciences and 10.13039/501100011176Peking Union Medical College, Shenzhen (E010122002). We would also like to thank Peng Liang & Minhao Sun (LC-Bio Technology CO., Ltd) for the assistance with WES data analysis.

## Author contributions

Conceptualization, L.G., D.W., J.Z., and J.W.; methodology, L.G., D.W., J.Z., J.W. X.H., and W.J.; investigation, L.G., D.W., J.Z., J.W., X.H. W.J., and W.G.; resources, W.G., J.T., and D.W.; data curation, J.Z., J.W., H.G., and G.K.; writing – original draft preparation, J.Z.; writing – review and editing, L.G., D.W., and J.W.; supervision, L.G. and D.W.; project administration, Z.Z., L.G., and D.W.; funding acquisition, Z.Z., L.G., and D.W.

## Declaration of interests

The authors declare no competing interests.

## STAR★Methods

### Key resources table

**Table:** 

REAGENT or RESOURCE	SOURCE	IDENTIFIER
**Antibodies**		

Rabbit anti-CK7	Proteintech	Cat# 17513-1-AP; PRID: AB_2134468
Rabbit anti-Ki67	Proteintech	Cat# 27309-1-AP; PRID: AB_2756525
Mouse anti-p40	Roche	Cat# 790-4950 PRID: AB_2935820
Rabbit anti-GATA3	Abclonal	Cat# A25955 PRID: AB_3719864
Rabbit anti-HER2	Abclonal	Cat# A21248 PRID: AB_3718566
Alexa Fluor™ 555 Donkey anti-Rabbit IgG	ThermoFisher	Cat# A-31572 PRID: AB_162543
Alexa Fluor™ 555 Goat anti-Mouse IgG	ThermoFisher	Cat# A-21422 PRID: AB_141822

**Biological samples**		

Human bladder cancer tissue	This paper	N/A
Human blood samples	This paper	N/A

**Chemicals, peptides, and recombinant proteins**		

Alexa Fluor™ 488 phalloidin	ThermoFisher	Cat# A12379
Alexa Fluor™ 555 phalloidin	ThermoFisher	Cat# A34055
MCFs	JINJIAHAO	Cat# CNF-H4
Gelatin from porcin skin	Sigma-Aldrich	Cat# V900863-500g
mTG	Ajinomoto Corporation	Cat# 170123A
MC-gel	This paper	N/A
Triton X-100	MaiXin	Cat# MVS-0066
Matrigel	Corning	Cat# 354234
Agarose	YEASEN	Cat# 10208ES60
Collagenase I	OriLeaf	Cat# S10053
Collagenase IV	OriLeaf	Cat# S10056
Y-27632 dihydrochloride	MedChemExoress	Cat# HY-10071
Triton X-100	Beyotime	Cat# P0096
DAPI solution	Beyotime	Cat# C1002
Anti-fade mounting medium	Beyotime	Cat# P0131
Cisplatin	UHNShangHai	Cat# 15663-27-1
Carboplatin	UHNShangHai	Cat# 41575-94-4
Oxaliplatin	UHNShangHai	Cat# 61825-94-3
Pirarubicin	MedChemExpress	Cat# HY-13725
Mitomycin C	AbMole Bioscience	Cat# M5791
Advanced DMEM/F12	Corning	Cat# 10-092-CV
1x HEPES	Gibco	Cat# 15630130
1x Glutamax	Gibco	Cat# 35050-061
1x penicillin/streptomycin	Beyotime	Cat# C0222
1x B27	Gibco	Cat# 17504044
1x Primocin	Invitrogen	Cat# ant-pm-2
N-acetyl-L-cysteine	MedChemExpress	Cat# HY-B0215
Wnt3a	GenScript	Cat# Z03815
Recombinant Noggin protein	GenScript	Cat# Z02753
Epidermal growth factor	GenScript	Cat# Z00333
Fibroblast growth factor 10	GenScript	Cat# Z03314
Nicotinamide	MedChemExpress	Cat# HY-B0150
SB202190	MedChemExpress	Cat# HY-10295
A83-01	MedChemExpress	Cat# HY-10432
8 μm pore transparent PET membranes	Falcon	Cat# 353097

**Critical commercial assays**		

DNeasy Blood & Tissue Kit	QIAGEN	Cat# 55204
Human Exome 2.0 Plus kit	Twist	Cat# 105036
CellTiter-Lumi™ Luminescent Cell Viability Assay kit	Beyotime	Cat# C0061S

**Deposited data**		

WES raw data	This paper	NCBI SRA (Short Read Archive): PRJNA1162232

**Experimental models: Cell lines**		

Patient-derived bladder cancer organoids	This paper	N/A
Patient-derived bladder cancer cells lines	This paper	N/A

**Software and algorithms**		

R version 4.3.1	R Project for Statistical Computing	https://cran.r-project.org; PRID: SCR_001905
IBM SPSS Statistics version 26	IBM SPSS Statistics	https://www.ibm.com/products/spss-statistics; RRID:SCR_016479
GraphPad Prism Software version 9.5	GraphPad Prism	https://www.graphpad.com; RRID:SCR_002798
ImageJ	National Institutes of Health	https://ImageJ.net/; PRID: SCR_003070
Clara Parabricks version 4.2.0	NVIDIA	https://docs.nvidia.com/clara/parabricks/4.2.0/index.html
Fastp version 0.23.1	N/A	https://github.com/OpenGene/fastp; PRID: RRID:SCR_016962
Mutect2 version 4.1	GATK	https://gatk.broadinstitute.org/hc/en-us/articles/360037593851-Mutect2; PRID: RRID:SCR_026692
Annovar	N/A	https://annovar.openbioinformatics.org/en/latest/; PRID: SCR_012821
FACETS	N/A	https://github.com/dariober/cnv_facets; PRID: SCR_026264
GISTIC version 2	N/A	https://broadinstitute.github.io/gistic2/; PRID: RRID:SCR_000151
Meskit	N/A	https://github.com/Niinleslie/MesKit/; PRID: SCR_020959
MutationalPatterns	N/A	https://github.com/UMCUGenetics/MutationalPatterns; PRID: SCR_024247

**Other**		

hg19	The UCSC Human Reference Genome	ftp://hgdownload.cse.ucsc.edu/goldenPath/hg19/bigZips/
1000G_phase1.indels.hg19.sites.simplified.vcf.gz,Mills_and_1000G_gold_standard.indels.hg19.sites.simplified.vcf.gz,dbsnp_138.hg19.simplified.vcf.gz	GATK BQSR	https://github.com/broadinstitute/gatk/tree/master/src/test/resources/large/variantRecalling

### Experimental model and study participant details

#### Tumor specimen collection

Bladder cancer tissues and blood samples, histologically verified as bladder cancer, were collected from patients undergoing surgical resection at Cancer Hospital & Shenzhen Hospital, Chinese Academy of Medical Sciences. The study participants consisted of 9 male and 5 female Asian subjects. The average age of the patients at the time of surgery was 66.36 years. Excised tumor tissues, ranging in size from 1 to 2.5 cm^3^, were promptly transported to the laboratory for processing. Preoperative blood samples were also collected one day before surgery for sequencing analysis. Detailed clinical information for the patients from whom the tumors were sourced is provided in Table S2. This research was approved by the Ethics Committee of the Cancer Institute and Hospital, Chinese Academy of Medical Sciences (approval number JS2023-28-1). The samples were obtained from patients who provided written informed consent for research prior to tissue collection.

#### Organoid culture

Initially, tumor samples were thoroughly rinsed with cold PBS twice and subsequently dissected into fine fragments using surgical blades. A segment of the tissue was allocated for histological assessment, while another was immediately frozen at −80°C for subsequent genetic evaluation. The remaining tissue fragments were further reduced to dimensions of 1–1.5 mm^3^, suitable for organoid culture. These finely minced tissue pieces were subsequently transferred to a 15 mL centrifuge tube and treated with 5 mL of a 3 mg/mL collagenase I (OriLeaf, Cat# S10053) solution for enzymatic digestion. After repeated shaking in a 37°C incubator for 1 h, the tissue was effectively dissociated into individual cells, a process halted by the addition of growth medium. The resulting cell suspension underwent filtration through a 100 μm sieve, followed by centrifugation at 1000*g* for 5 min. Remove the supernatant and rinse the cell pellet with organoid culture medium for two times. Resuspend the cells and count them. Part of the cells were stored in a cryoprotectant medium containing 10% dimethyl sulfoxide (DMSO) (MPbio, Cat#196055), 20% fetal bovine serum (FBS) (Gibco, Cat#A5256701), and 70% Advanced DMEM/F12 (Corning, Cat#10-092-CV) at −80°C for later drug sensitivity experiments. The remaining primary tumor cells were then resuspended in a 4% gelatin solution (Sigma-Aldrich, Cat# V900863-500g).^47^ Subsequently, the cell suspension was carefully dispensed into a 6-well plate, with each well receiving 10 drops of a mixture, each approximately 20 μL in volume and containing roughly 20,000 cells, to ensure uniform distribution. Once the synthetic hydrogel had been given time to solidify at 37°C within an incubator maintained at 5% CO_2_, 2 mL of culture medium was then introduced into each well. The organoid culture medium, as detailed in Table S1, was meticulously replaced every three days for the duration of the incubation.

After 14 to 21 days of cultivation in organoid form, passaging was typically performed when organoids, depending on their growth rates and morphological characteristics, reached a diameter of approximately 100–200 μm—a size that balances optimal viability and proliferative capacity. Subculturing was carried out at dilutions of 1:2 to 1:3. During the subculturing process, the cell-gel composite was divided into minute segments using a cell dissection instrument and then spun down at 1000g for a period of 3 min. After the supernatant was discarded, the pellet was treated with a 2 mg/mL solution of collagenase IV (OriLeaf, Cat#S10056) and incubated at 37°C for a duration ranging from 5 to 10 min, with 10 μM Y-27632 dihydrochloride (MedChemExoress, Cat# HY-10071). Centrifugation of the organoids was performed at 200g for a duration of 5 min, followed by a thorough wash with the culture medium and then subjected to centrifugation again. For the purpose of preservation, the organoids were carefully isolated from the gel matrix and subjected to cryopreservation. This process involved immersion in a cell freezing medium enriched with 10 μM Y-27632 dihydrochloride, followed by storage under liquid nitrogen conditions. For the selection of experimental samples, those exhibiting stable growth during long-term culture and possessing sufficient tissue for multiple analyses were prioritized. The passage numbers for the experimental samples were listed in Table S3.

#### Primary bladder cancer cell lines

For primary suspension cell preparation, cells were removed from the −80°C freezer and immediately thawed in a 37°C water bath. Upon thawing, the cell suspension was transferred to a centrifuge tube containing 10 mL Advanced DMEM/F12. The suspension was then centrifuged at 300g for 5 min to remove the supernatant. The resulting cell pellet was resuspended in Advanced DMEM/F12. The cell suspension was gently mixed to ensure uniform distribution and subsequently seeded into suitable culture vessels. The cells were placed in a 37°C incubator with 5% CO_2_ for cultivation. After 24 h, cell morphology and viability were assessed under a microscope to ensure proper recovery. Cell viability was determined by live cell counting.

### Method details

#### Biosynthetic hydrogel preparation

To ensure consistency, commercially acquired MCFs (JINJIAHAO, Cat#CNF-H4) were initially homogenized at 4°C using a tissue grinder operating at 6.5 m/s, with a cycle program of 1 min on and 1 min off for a total of seven cycles. Following the homogenization process, the MCFs were sterilized by immersion in 75% ethanol for 1 h, prior to the fabrication of the bio-synthetic hydrogel. The MCFs were then thoroughly rinsed with sterile deionized water 4–5 times and stored at −80 °C. A 4% gelatin solution was then prepared by heating sterile water and mixing it with porcine skin gelatin (Sigma-Aldrich, Cat# V900863-500g) until the solution reached 50°C. Without delay, the solution underwent filtration through a 0.22-micron mesh, ensuring the elimination of particulate matter. The gelatin solution was then combined with the previously thawed MCFs at a 0.1% concentration. To facilitate the cross-linking of gelatin, mTG, a transglutaminase enzyme from *Streptomyces mobaraensis* (Ajinomoto Corporation, Cat#170123A), was incorporated into the solution. The mTG enzyme was used uniformly at an activity level of 20,000 U/mg. The MC-gel was prepared following the methodology detailed in our previous report.^47^

#### Scanning electron microscopy

To preserve their porous structures, the MCFs hydrogel and control hydrogel samples were first rapidly frozen in liquid nitrogen for 10 min. They were then freeze-dried for 24 h under a vacuum pressure of 2 Pa to ensure complete dehydration. Once dried, the gels were carefully sectioned with a surgical blade to prepare them for further analysis. These sections were mounted onto aluminum stubs and subsequently coated with a thin layer of gold using sputter deposition, which enhanced their conductivity for imaging purposes. The porous morphology was examined using a scanning electron microscope (HITACHI Regulus 8100) at 20 kV, beginning with a magnification of 100×. For quantitative analysis, pore diameters were measured in 60 randomly selected pores from each group, providing a comprehensive characterization of the structural properties of the gels.

#### H&E analysis

Fresh tissue samples, designated for histological examination, were immersed in a 4% paraformaldehyde (PFA) solution for 24 h to ensure fixation. Subsequently, the tissues underwent dehydration through a gradient of increasing ethanol concentrations. Following dehydration, the tissues were subjected to clarification in xylene and then processed for paraffin embedding. Simultaneously, organoids cultured within a gel matrix were also fixed in a 4% paraformaldehyde solution for a duration of 24 h. These were subsequently encapsulated within 1.5 mL centrifuge tubes containing 2% agarose (YEASEN, Cat#10208ES60). The organoids then underwent a similar process of dehydration through an ascending gradient of ethanol concentrations, clarification in xylene, and paraffin embedding. Both the embedded tissues and organoids were sectioned to a precise thickness of 4 μm, mounted onto glass slides, and dried overnight at 60°C. The paraffin-embedded sections were then subjected to deparaffinization using xylene and a subsequent rehydration through a gradient of decreasing concentrations of ethanol. Once these steps were completed, the sections were prepared for staining procedures.

#### If staining

To further examine the morphology of tissues and organoids, immunofluorescence (IF) staining was conducted. Paraffin sections were obtained following the H&E analysis procedure, which included deparaffinization and hydration. Antigen retrieval was performed by heating the sections in a pressure cooker with citrate solution adjusted to a pH of 6.0. The sections were then treated with 0.1% Triton X-100 solution (MaiXin, Cat#MVS-0066) at 37°C for 15 min to enhance antibody penetration, followed by rinsing with PBS. Subsequently, a blocking step was carried out using 3% BSA at 37°C for 1 h to reduce non-specific binding, after which the sections were rinsed again with PBS. Primary antibodies, including cytokeratin 7 (CK7, Proteintech, Cat#17513-1-AP), Ki-67 (Proteintech, Cat#27309-1-AP), p40 (Roche, Cat#790–4950), GATA3 (Abclonal, Cat#A25955), and HER2 (Abclonal, Cat#A21248) were diluted in TBS buffer containing 1% BSA and applied to the tissue and organoid sections for overnight incubation at 4°C. F-actin was labeled using phalloidin conjugates (ThermoFisher, Cat#A12379; Cat#A34055). After a room temperature rewarming step, unbound antibodies were thoroughly washed away. Fluorescently labeled secondary antibodies, specific to the species of origin of the primary antibodies, Donkey anti-Rabbit IgG (ThermoFisher, Cat#A-31572) and Goat anti-Mouse IgG (ThermoFisher, Cat#A-21422), were then applied and incubated at 37°C in the dark for 1 h. Following incubation, the slides were washed again using TBST and TBS buffers to remove excess secondary antibodies. Nuclear staining was performed with DAPI (Beyotime, Cat#C1002) for 10 min, after which the slides were subjected to a final series of washes with TBST and TBS buffers to remove excess DAPI. Finally, the slides were mounted using an anti-fade mounting medium (Beyotime, Cat#P0131) and the stained sections were visualized using a confocal microscope (model LSM900) produced by Zeiss Microsystems Corporation.

#### DNA isolation and examination in whole exome sequencing studies

The whole exome sequencing samples, including six BCOs, corresponding bladder tissues, and matched pre-operative blood, were prepared for WES analysis. Total DNA was extracted using the DNeasy Blood & Tissue Kit (QIAGEN, Cat#55204) to ensure high purity and integrity. This was followed by the meticulous fragmentation of the DNA, executed with precision using the Covaris M220 Focused-ultrasonicator, a critical step for the subsequent library preparation. For the targeted enrichment of exonic regions, the Human Exome 2.0 Plus Kit (Twist, Cat#105036) was meticulously applied, adhering to the stringent protocols provided by the manufacturer. The resultant libraries were then subjected to high-throughput sequencing on the Illumina NovaSeq 6000 platform at LC-Bio Technology Co., Ltd, Hangzhou, China, yielding paired-end reads of 150 base pairs.

Quality control was rigorously enforced at the initial stage, with the fastp software suite employed to meticulously filter out sequences of inferior quality.^76^ The Burrows-Wheeler Aligner (BWA) was then engaged to map the reads to the hg19 reference genome, a fundamental step for accurate alignment.^77^ Post-alignment, the Picard tools were adeptly utilized to detect and flag any duplicate reads within the BAM files, ensuring the reliability of the subsequent analysis. Further refinement was achieved through local realignment to correct any misalignments, especially in the vicinity of indels, followed by a thorough recalibration of base quality scores to mitigate potential biases. The identification of somatic variants, including SNVs and inDels, was conducted using Mutect2, which provided a robust framework for variant calling.^78^ To enrich the dataset with biological context, ANNOVAR was strategically applied for comprehensive annotation.^79^ The FACETS software was then employed for the detection of copy number variations, a significant aspect of the genomic analysis.^80^ The GC content of the sequences was thoughtfully considered for normalizing the genome-wide read coverage, allowing for a more accurate assessment of copy number differences between tumor and normal samples.

#### *In vitro* drug studies

Cisplatin (UHNShangHai, Cat#15663-27-1), carboplatin (UHNShangHai, Cat#41575-94-4), and oxaliplatin (UHNShangHai, Cat#61825-94-3) were selected for the drug experiments. The drugs were reconstituted in Advanced DMEM/F12 following the manufacturer’s instructions to achieve a gradient of concentrations: 10, 2.5, 0.625, 0.15625, and 0.0390625 μM. Drug sensitivity experiments were conducted on both organoids and their corresponding primary suspension cells under identical conditions to evaluate their responses to the treatments.

To validate the consistency of the organoid model’s response to patient drug reactions, organoids BCO-3, BCO-5, BCO-9, and BCO-11, which were derived from patients treated with intravesical agents pirarubicin (MedChemExpress, Cat#HY-13725) or mitomycin C (AbMole Bioscience, Cat#M5791), were utilized. Following the manufacturer’s guidelines, the solutions of pirarubicin and mitomycin C were prepared in Advanced DMEM/F12 at working concentration of 5 μg/mL (pirarubicin) and 5 μmol/L (mitomycin C). Subsequently, bladder cancer organoids were dissociated into single cells, enumerated, and seeded at a density of 10,000 cells per well in 96-well plates, with each condition replicated thrice. Upon 7 to 10 days of culture, the cells underwent a 3-to-5-day treatment with the drugs.

Viability of the organoids was determined by employing the CellTiter-Lumi™ Luminescent Cell Viability Assay kit (Beyotime, Cat#C0061S), strictly following the protocol provided by the manufacturer. To account for variability between culture plates, control groups were normalized for activity: water or culture medium-treated wells served as negative controls with 0% activity, while untreated wells were set to 100%. To ensure accurate drug sensitivity assessment, activity rates were calculated from triplicate samples.

#### Migration assay

The migratory potential of organoids was gauged using Transwell migration assays, which were conducted using Transwell chambers with 8-μm pore transparent PET membranes (Falcon, Cat#353097) in a 24-well plate. A volume of 200 μL of serum-devoid DMEM/F12 was used to suspend the cells for seeding into the upper compartment of each insert, while the lower compartment was filled with 600–800 μL of medium supplemented with a chemical attractant. Organoids, following dissociation into individual cells at a concentration of 1 × 10ˆ5 cells/mL, were suspended in the 200 μL serum-devoid DMEM/F12 and then transferred to the upper compartment of the Transwell apparatus. Subsequently, the assembly was placed within an incubation environment set at 37°C and infused with 5% CO_2_ for a duration of 24–48 h to facilitate cell migration.

Upon completion of the incubation phase, the inserts were carefully extracted. The upper compartment was rinsed with sterile PBS. Non-migrated cells on the upper membrane surface were then gently removed with a cotton swab. The cells that had traversed to the underside of the membrane were subjected to fixation using a 4% PFA solution for a period ranging from 10 to 15 min. Post-fixation, the cells were rinsed once more with PBS prior to being subjected to DAPI staining. The migration of cells across the lower membrane surface was scrutinized under microscopic examination, with photographic documentation taken for analysis. The enumeration of migrated cells across various visual fields was carried out, culminating in the computation of an average count. This quantitative data were subsequently analyzed to ascertain the migratory efficacy of the organoids. Each condition was repeated at least four times to ensure accuracy and reproducibility.

#### Invasion experiment

The invasive potential of organoids was evaluated using Transwell invasion assays, employing transwell inserts with 8-μm pore transparent PET membranes. A 50 mL layer of 1 : 8 diluted Matrigel (Corning, Cat#354234) was applied to the insert membranes to simulate the extracellular matrix, following which the inserts were positioned in a 24-well plate and incubated at 37°C to facilitate gel polymerization for 1 h. Simultaneously, the organoids were processed into a homogeneous single-cell suspension at a density of 1 × 10ˆ5 cells/mL, with 200 μL of serum-devoid DMEM/F12 being introduced into the upper chamber of the transwell. The lower chamber was supplemented with 600–800 μL of medium laced with a chemoattractant. The assembly was then subjected to an incubation period of 24–48 h within a 37°C, 5% CO_2_ environment to promote cell invasion.

Upon completion of the incubation, the inserts were extracted and the upper chamber was delicately rinsed with sterile PBS to remove cells that failed to penetrate the Matrigel. The cells that had traversed through the Matrigel and adhered to the lower surface of the membrane were fixed with 4% PFA for 15 min. Post-fixation, the cells were rinsed with PBS and subjected to DAPI staining to highlight the nuclei. The upper membrane surface was cleared of non-invading cells using a cotton swab. The lower surface of the membrane, now displaying invaded cells, was scrutinized under a microscope, with images being documented for subsequent analysis. A quantification of invaded cells was performed across various visual fields, and the mean count was ascertained. This aggregation of data was subsequently analyzed statistically to delineate the invasive behavior of the organoids, adhering to the methodological standards requisite for scholarly discourse.

#### Adhesion assay after drug trails

Organoids treated with pirarubicin or mitomycin C were dissociated into single-cell suspensions and seeded onto extracellular matrix-coated microplates. The cell suspension (1 × 10ˆ5 cells/mL) was seeded into each well of the microplate with 200 μL per well, allowing the cells to adhere to the matrix. The cells were incubated for 24 h at 37°C with 5% CO_2_ to facilitate matrix adhesion. After incubation, non-adherent cells were removed by gentle shaking or washing. In parallel wells, cell viability was assessed using the CellTiter-Lumi™ Luminescent Cell Viability Assay kit. The remaining wells were used for cell fixation, where adherent cells were fixed with 4% PFA for 15 min. After fixation, the cells were washed with PBS and stained with DAPI and phalloidin to label F-actin. The morphology and distribution of the adherent cells were examined using optical and confocal microscopy.

### Quantification and statistical analysis

The experimental results are presented as the mean ± standard deviation (SD) for normally distributed data, or as the median with interquartile range (IQR) for data with small sample (*n* < 10) or non-normally distributed variables. Statistical analysis was performed using GraphPad Prism Software (Version 9.5) and IBM SPSS Statistics (Version 26). Data visualization was performed using R (Version 4.3.1) and GraphPad Prism (Version 9.5). To assess the differences between two groups, an unpaired two-tailed Student’s *t* test was used, while one-way Kruskal-Wallis test with Dunn’s post hoc test was applied for comparisons among three groups. Significance in statistics was denoted by a *p*-value below the threshold of 0.05. For figure legends, statistical significance is indicated by asterisks: ∗*p* < 0.05, ∗∗*p* < 0.01, and ∗∗∗*p* < 0.001.

## Supplemental information

Document S1. Figures S1–S3 and Tables S1–S4

Table S5. Compendium cancer genes, related to Figure 4

## References

[1] Siegel R.L., Giaquinto A.N., Jemal A. (2024). Cancer statistics. CA Cancer J. Clin..

[2] Stecca C., Abdeljalil O., Sridhar S.S. (2021). Metastatic Urothelial Cancer: a rapidly changing treatment landscape. Ther. Adv. Med. Oncol..

[3] Claps F., Pavan N., Ongaro L., Tierno D., Grassi G., Trombetta C., Tulone G., Simonato A., Bartoletti R., Mertens L.S. (2023). BCG-Unresponsive Non-Muscle-Invasive Bladder Cancer: Current Treatment Landscape and Novel Emerging Molecular Targets. Int. J. Mol. Sci..

[4] Teoh J.Y.C., Kamat A.M., Black P.C., Grivas P., Shariat S.F., Babjuk M. (2022). Recurrence mechanisms of non-muscle-invasive bladder cancer - a clinical perspective. Nat. Rev. Urol..

[5] Zargar H., Espiritu P.N., Fairey A.S., Mertens L.S., Dinney C.P., Mir M.C., Krabbe L.M., Cookson M.S., Jacobsen N.E., Gandhi N.M. (2015). Multicenter assessment of neoadjuvant chemotherapy for muscle-invasive bladder cancer. Eur. Urol..

[6] Witjes J.A., Bruins H.M., Cathomas R., Compérat E.M., Cowan N.C., Gakis G., Hernández V., Linares Espinós E., Lorch A., Neuzillet Y. (2021). European Association of Urology Guidelines on Muscle-invasive and Metastatic Bladder Cancer: Summary of the 2020 Guidelines. Eur. Urol..

[7] Wang X., Lu Y., Wang W., Wang Q., Liang J., Fan Y., Zhang X. (2020). Effect of different aged cartilage ECM on chondrogenesis of BMSCs in vitro and in vivo. Regen. Biomater..

[8] Ergun C., Parmaksiz M., Vurat M.T., Elçin A.E., Elçin Y.M. (2022). Decellularized liver ECM-based 3D scaffolds: Compositional, physical, chemical, rheological, thermal, mechanical, and in vitro biological evaluations. Int. J. Biol. Macromol..

[9] Horvath P., Aulner N., Bickle M., Davies A.M., Nery E.D., Ebner D., Montoya M.C., Östling P., Pietiäinen V., Price L.S. (2016). Screening out irrelevant cell-based models of disease. Nat. Rev. Drug Discov..

[10] Ben-David U., Ha G., Tseng Y.Y., Greenwald N.F., Oh C., Shih J., McFarland J.M., Wong B., Boehm J.S., Beroukhim R., Golub T.R. (2017). Patient-derived xenografts undergo mouse-specific tumor evolution. Nat. Genet..

[11] Byrne A.T., Alférez D.G., Amant F., Annibali D., Arribas J., Biankin A.V., Bruna A., Budinská E., Caldas C., Chang D.K. (2017). Interrogating open issues in cancer medicine with patient-derived xenografts. Nat. Rev. Cancer.

[12] Xu H., Jiao D., Liu A., Wu K. (2022). Tumor organoids: applications in cancer modeling and potentials in precision medicine. J. Hematol. Oncol..

[13] Xu S., Tan S., Guo L. (2023). Patient-Derived Organoids as a Promising Tool for Multimodal Management of Sarcomas. Cancers.

[14] Kim M., Mun H., Sung C.O., Cho E.J., Jeon H.J., Chun S.M., Jung D.J., Shin T.H., Jeong G.S., Kim D.K. (2019). Patient-derived lung cancer organoids as in vitro cancer models for therapeutic screening. Nat. Commun..

[15] Shi R., Radulovich N., Ng C., Liu N., Notsuda H., Cabanero M., Martins-Filho S.N., Raghavan V., Li Q., Mer A.S. (2020). Organoid Cultures as Preclinical Models of Non-Small Cell Lung Cancer. Clin. Cancer Res..

[16] Sui Z., Wu X., Wang J., Tan S., Zhao C., Yu Z., Wu C., Wang X., Guo L. (2025). Mesenchymal stromal cells promote the formation of lung cancer organoids via Kindlin-2. Stem Cell Res. Ther..

[17] Calandrini C., Schutgens F., Oka R., Margaritis T., Candelli T., Mathijsen L., Ammerlaan C., van Ineveld R.L., Derakhshan S., de Haan S. (2020). An organoid biobank for childhood kidney cancers that captures disease and tissue heterogeneity. Nat. Commun..

[18] Esser L.K., Branchi V., Leonardelli S., Pelusi N., Simon A.G., Klümper N., Ellinger J., Hauser S., Gonzalez-Carmona M.A., Ritter M. (2020). Cultivation of Clear Cell Renal Cell Carcinoma Patient-Derived Organoids in an Air-Liquid Interface System as a Tool for Studying Individualized Therapy. Front. Oncol..

[19] Lee S.H., Hu W., Matulay J.T., Silva M.V., Owczarek T.B., Kim K., Chua C.W., Barlow L.J., Kandoth C., Williams A.B. (2018). Tumor evolution and drug response in patient-derived organoid models of bladder cancer. Cell.

[20] Minoli M., Cantore T., Hanhart D., Kiener M., Fedrizzi T., La Manna F., Karkampouna S., Chouvardas P., Genitsch V., Rodriguez-Calero A. (2023). Bladder cancer organoids as a functional system to model different disease stages and therapy response. Nat. Commun..

[21] Rosenbluth J.M., Schackmann R.C.J., Gray G.K., Selfors L.M., Li C.M.C., Boedicker M., Kuiken H.J., Richardson A., Brock J., Garber J. (2020). Organoid cultures from normal and cancer-prone human breast tissues preserve complex epithelial lineages. Nat. Commun..

[22] Guillen K.P., Fujita M., Butterfield A.J., Scherer S.D., Bailey M.H., Chu Z., DeRose Y.S., Zhao L., Cortes-Sanchez E., Yang C.H. (2022). A human breast cancer-derived xenograft and organoid platform for drug discovery and precision oncology. Nat. Cancer.

[23] Sato T., Stange D.E., Ferrante M., Vries R.G.J., Van Es J.H., Van den Brink S., Van Houdt W.J., Pronk A., Van Gorp J., Siersema P.D., Clevers H. (2011). Long-term expansion of epithelial organoids from human colon, adenoma, adenocarcinoma, and Barrett's epithelium. Gastroenterology.

[24] Yan H.H.N., Siu H.C., Ho S.L., Yue S.S.K., Gao Y., Tsui W.Y., Chan D., Chan A.S., Wong J.W.H., Man A.H.Y. (2020). Organoid cultures of early-onset colorectal cancers reveal distinct and rare genetic profiles. Gut.

[25] Gao D., Vela I., Sboner A., Iaquinta P.J., Karthaus W.R., Gopalan A., Dowling C., Wanjala J.N., Undvall E.A., Arora V.K. (2014). Organoid cultures derived from patients with advanced prostate cancer. Cell.

[26] Drost J., Karthaus W.R., Gao D., Driehuis E., Sawyers C.L., Chen Y., Clevers H. (2016). Organoid culture systems for prostate epithelial and cancer tissue. Nat. Protoc..

[27] Nia H.T., Munn L.L., Jain R.K. (2020). Physical traits of cancer. Science.

[28] Meng F., Shen C., Yang L., Ni C., Huang J., Lin K., Cao Z., Xu S., Cui W., Wang X. (2022). Mechanical stretching boosts expansion and regeneration of intestinal organoids through fueling stem cell self-renewal. Cell Regen..

[29] Discher D.E., Janmey P., Wang Y.L. (2005). Tissue cells feel and respond to the stiffness of their substrate. Science.

[30] Hynes R.O. (2009). The extracellular matrix: not just pretty fibrils. Science.

[31] Iuliano J.N., Kutscha P.D., Biderman N.J., Subbaram S., Groves T.R., Tenenbaum S.A., Hempel N. (2015). Metastatic bladder cancer cells distinctively sense and respond to physical cues of collagen fibril-mimetic nanotopography. Exp. Biol. Med..

[32] Qiu S., Deng L., Liao X., Nie L., Qi F., Jin K., Tu X., Zheng X., Li J., Liu L. (2019). Tumor-associated macrophages promote bladder tumor growth through PI3K/AKT signal induced by collagen. Cancer Sci..

[33] Zhu H., Chen H., Wang J., Zhou L., Liu S. (2019). Collagen stiffness promoted non-muscle-invasive bladder cancer progression to muscle-invasive bladder cancer. OncoTargets Ther..

[34] Ingenwerth M., Nyirády P., Hadaschik B., Szarvas T., Reis H. (2022). The prognostic value of cytokeratin and extracellular collagen expression in urinary bladder cancer. Curr. Mol. Med..

[35] Huang Y., Fang S., Xie W., Zou Y., Zhuo H., Shen G., Zhou H., Mao C., Lai C., Kong J., Fan X. (2025). Collagen gene signature in the tumor microenvironment predicts survival and guides prognosis in bladder cancer. Discov. Oncol..

[36] Pointer K.B., Clark P.A., Schroeder A.B., Salamat M.S., Eliceiri K.W., Kuo J.S. (2017). Association of collagen architecture with glioblastoma patient survival. J. Neurosurg..

[37] Penet M.-F., Kakkad S., Pathak A.P., Krishnamachary B., Mironchik Y., Raman V., Solaiyappan M., Bhujwalla Z.M. (2017). Structure and function of a prostate Cancer dissemination–permissive extracellular matrix. Clin. Cancer Res..

[38] Acerbi I., Cassereau L., Dean I., Shi Q., Au A., Park C., Chen Y.Y., Liphardt J., Hwang E.S., Weaver V.M. (2015). Human breast cancer invasion and aggression correlates with ECM stiffening and immune cell infiltration. Integr. Biol..

[39] Brightman A.O., Rajwa B.P., Sturgis J.E., McCallister M.E., Robinson J.P., Voytik-Harbin S.L. (2000). Time-lapse confocal reflection microscopy of collagen fibrillogenesis and extracellular matrix assembly in vitro. Biopolymers.

[40] Gong X., Kulwatno J., Mills K.L. (2020). Rapid fabrication of collagen bundles mimicking tumor-associated collagen architectures. Acta Biomater..

[41] Kai F., Drain A.P., Weaver V.M. (2019). The extracellular matrix modulates the metastatic journey. Dev. Cell.

[42] Conklin M.W., Keely P.J. (2012). Why the stroma matters in breast cancer: insights into breast cancer patient outcomes through the examination of stromal biomarkers. Cell Adh. Migr..

[43] Di Lullo G.A., Sweeney S.M., Korkko J., Ala-Kokko L., San Antonio J.D. (2002). Mapping the ligand-binding sites and disease-associated mutations on the most abundant protein in the human, type I collagen. J. Biol. Chem..

[44] Ray A., Lee O., Win Z., Edwards R.M., Alford P.W., Kim D.H., Provenzano P.P. (2017). Anisotropic forces from spatially constrained focal adhesions mediate contact guidance directed cell migration. Nat. Commun..

[45] Tabdanov E.D., Puram V., Zhovmer A., Provenzano P.P. (2018). Microtubule-actomyosin mechanical cooperation during contact guidance sensing. Cell Rep..

[46] Liu C., Nguyen R.Y., Pizzurro G.A., Zhang X., Gong X., Martinez A.R., Mak M. (2023). Self-assembly of mesoscale collagen architectures and applications in 3D cell migration. Acta Biomater..

[47] Wang J., Sui Z., Huang W., Yu Z., Guo L. (2024). Biomimetic hydrogels with mesoscale collagen architecture for patient-derived tumor organoids culture. Bioact. Mater..

[48] Prince E., Cruickshank J., Ba-Alawi W., Hodgson K., Haight J., Tobin C., Wakeman A., Avoulov A., Topolskaia V., Elliott M.J. (2022). Biomimetic hydrogel supports initiation and growth of patient-derived breast tumor organoids. Nat. Commun..

[49] Amara C., El Mahdi A., Akman P.K., Medimagh R., Tornuk F., Khwaldia K. (2023). Use of cellulose microfibers from olive pomace to reinforce green composites for sustainable packaging applications. Food Sci. Nutr..

[50] Li Z., Lin C., Zhao L., Zhou L., Pan X., Quan J., Peng X., Li W., Li H., Xu J. (2018). Oncogene miR-187-5p is associated with cellular proliferation, migration, invasion, apoptosis and an increased risk of recurrence in bladder cancer. Biomedicine & pharmacotherapy = Biomedecine & pharmacotherapie.

[51] Zhou C., Wang J., Zhang Y., Zhou Z., Wu C., Wang L., Guo L. (2025). Cellulose microfiber-mediated mesoscale architecture promotes the expansion of patient-derived lung cancer organoids while preserving their malignant characteristics. J. Mater. Chem. B.

[52] Cannataro V.L., Mandell J.D., Townsend J.P. (2022). Attribution of cancer origins to endogenous, exogenous, and preventable mutational processes. Mol. Biol. Evol..

[53] Lalazar G., Simon S.M. (2018). Fibrolamellar carcinoma: recent advances and unresolved questions on the molecular mechanisms. Semin. Liver Dis..

[54] Zhao H., Qiao J., Cao L. (2025). Bioactive ligands targeting ectopic olfactory receptors: Implications for therapeutic strategies. Br. J. Pharmacol..

[55] Leow M.K.S., Ang J., Bi X., Koh E.T., McFarlane C. (2023). Alterations in SAMD9, AHSG, FRG2C, and FGFR4 genes in a case of late-onset massive tumoral calcinosis. AACE Clin. Case Rep..

[56] Liu S., He Y., Li S., Gao X., Yang F. (2023). Kinesin family member 3A induces related diseases via wingless-related integration site/β-catenin signaling pathway. Sci. Prog..

[57] Cinar B., Alp E., Al-Mathkour M., Boston A., Dwead A., Khazaw K., Gregory A. (2021). The Hippo pathway: an emerging role in urologic cancers. Am. J. Clin. Exp. Urol..

[58] Aquila L., Ohm J., Woloszynska-Read A. (2018). The role of STAG2 in bladder cancer. Pharmacol. Res..

[59] Conde M., Frew I.J. (2022). Therapeutic significance of ARID1A mutation in bladder cancer. Neoplasia.

[60] Zhou L., Wang B., Zhang Y., Yao K., Liu B. (2021). Silencing circ-BIRC6 inhibits the proliferation, invasion, migration and epithelial-mesenchymal transition of bladder cancer cells by targeting the miR-495-3p/XBP1 signaling axis. Mol. Med. Rep..

[61] Ding B., Yan L., Zhang Y., Wang Z., Zhang Y., Xia D., Ye Z., Xu H. (2019). Analysis of the role of mutations in the KMT2D histone lysine methyltransferase in bladder cancer. FEBS open bio.

[62] Kompier L.C., Lurkin I., van der Aa M.N.M., van Rhijn B.W.G., van der Kwast T.H., Zwarthoff E.C. (2010). FGFR3, HRAS, KRAS, NRAS and PIK3CA mutations in bladder cancer and their potential as biomarkers for surveillance and therapy. PLoS One.

[63] Pérez-Montiel M.D., Cerrato-Izaguirre D., Sánchez-Pérez Y. (2023). Mutational Landscape of Bladder Cancer in Mexican Patients: KMT2D Mutations and chr11q15.5 Amplifications Are Associated with Muscle Invasion. Int. J. Mol. Sci..

[64] Langbein S., Szakacs O., Wilhelm M., Sukosd F., Weber S., Jauch A., Lopez Beltran A., Alken P., Kälble T., Kovacs G. (2002). Alteration of the LRP1B gene region is associated with high grade of urothelial cancer. Lab. Invest..

[65] Huang L., Peng Y., Zhong G., Xie W., Dong W., Wang B., Chen X., Gu P., He W., Wu S. (2015). PBRM1 suppresses bladder cancer by cyclin B1 induced cell cycle arrest. Oncotarget.

[66] Solomon D.A., Kim J.S., Bondaruk J., Shariat S.F., Wang Z.F., Elkahloun A.G., Ozawa T., Gerard J., Zhuang D., Zhang S. (2013). Frequent truncating mutations of STAG2 in bladder cancer. Nat. Genet..

[67] Mao Z., Gao F., Sun T., Xiao Y., Wu J., Xiao Y., Chu H., Wu D., Du M., Zheng R., Zhang Z. (2024). RB1 Mutations Induce Smoking-Related Bladder Cancer by Modulating the Cytochrome P450 Pathway. Environ. Toxicol..

[68] Karni-Schmidt O., Castillo-Martin M., Shen T.H., Gladoun N., Domingo-Domenech J., Sanchez-Carbayo M., Li Y., Lowe S., Prives C., Cordon-Cardo C. (2011). Distinct expression profiles of p63 variants during urothelial development and bladder cancer progression. Am. J. Pathol..

[69] Prip F., Lamy P., Lindskrog S.V., Strandgaard T., Nordentoft I., Birkenkamp-Demtröder K., Birkbak N.J., Kristjánsdóttir N., Kjær A., Andreasen T.G. (2025). Comprehensive genomic characterization of early-stage bladder cancer. Nat. Genet..

[70] Robertson A.G., Kim J., Al-Ahmadie H., Bellmunt J., Guo G., Cherniack A.D., Hinoue T., Laird P.W., Hoadley K.A., Akbani R. (2018). Comprehensive Molecular Characterization of Muscle-Invasive Bladder Cancer. Cell.

[71] Revah O., Gore F., Kelley K.W., Andersen J., Sakai N., Chen X., Li M.Y., Birey F., Yang X., Saw N.L. (2022). Maturation and circuit integration of transplanted human cortical organoids. Nature.

[72] Nelson C.M., Bissell M.J. (2006). Of extracellular matrix, scaffolds, and signaling: tissue architecture regulates development, homeostasis, and cancer. Annu. Rev. Cell Dev. Biol..

[73] Hogrebe N.J., Reinhardt J.W., Gooch K.J. (2017). Biomaterial microarchitecture: a potent regulator of individual cell behavior and multicellular organization. J. Biomed. Mater. Res..

[74] Zhao C., Xiao Y., Ling S., Pei Y., Ren J. (2021). Structure of Collagen. Methods Mol. Biol..

[75] Akrida I., Papadaki H. (2023). Adipokines and epithelial-mesenchymal transition (EMT) in cancer. Mol. Cell. Biochem..

[76] Chen S., Zhou Y., Chen Y., Gu J. (2018). fastp: an ultra-fast all-in-one FASTQ preprocessor. Bioinformatics.

[77] Li H., Durbin R. (2009). Fast and accurate short read alignment with Burrows-Wheeler transform. Bioinformatics (Oxford, England).

[78] Cibulskis K., Lawrence M.S., Carter S.L., Sivachenko A., Jaffe D., Sougnez C., Gabriel S., Meyerson M., Lander E.S., Getz G. (2013). Sensitive detection of somatic point mutations in impure and heterogeneous cancer samples. Nat. Biotechnol..

[79] Wang K., Li M., Hakonarson H. (2010). ANNOVAR: functional annotation of genetic variants from high-throughput sequencing data. Nucleic Acids Res..

[80] Shen R., Seshan V.E. (2016). FACETS: allele-specific copy number and clonal heterogeneity analysis tool for high-throughput DNA sequencing. Nucleic Acids Res..

