# Supplementary material for: Biomimetic hydrogel for the construction of patient-derived bladder cancer organoids with aggressive growth
Source: iScience. 2026 Jan 27;29(2):114786. doi: 10.1016/j.isci.2026.114786 (PMC12907049; doi:10.1016/j.isci.2026.114786)
Supplement: Document S1. Figures S1–S3 and Tables S1–S4 [file mmc1.pdf]

iScience, Volume *29*

Supplemental information

Biomimetic hydrogel for the construction of patient-derived bladder cancer organoids with aggressive growth

Jin Zhang, Jiaxin Wang, Xiaofeng Hu, Wei Jia, Ziyuan Zhou, Gaohaer Kadeerhan, Wenmin Guo, Jun Tian, Hong Guo, Ling Guo, and Dongwen Wang

Supplemental Information

Supplemental Figures

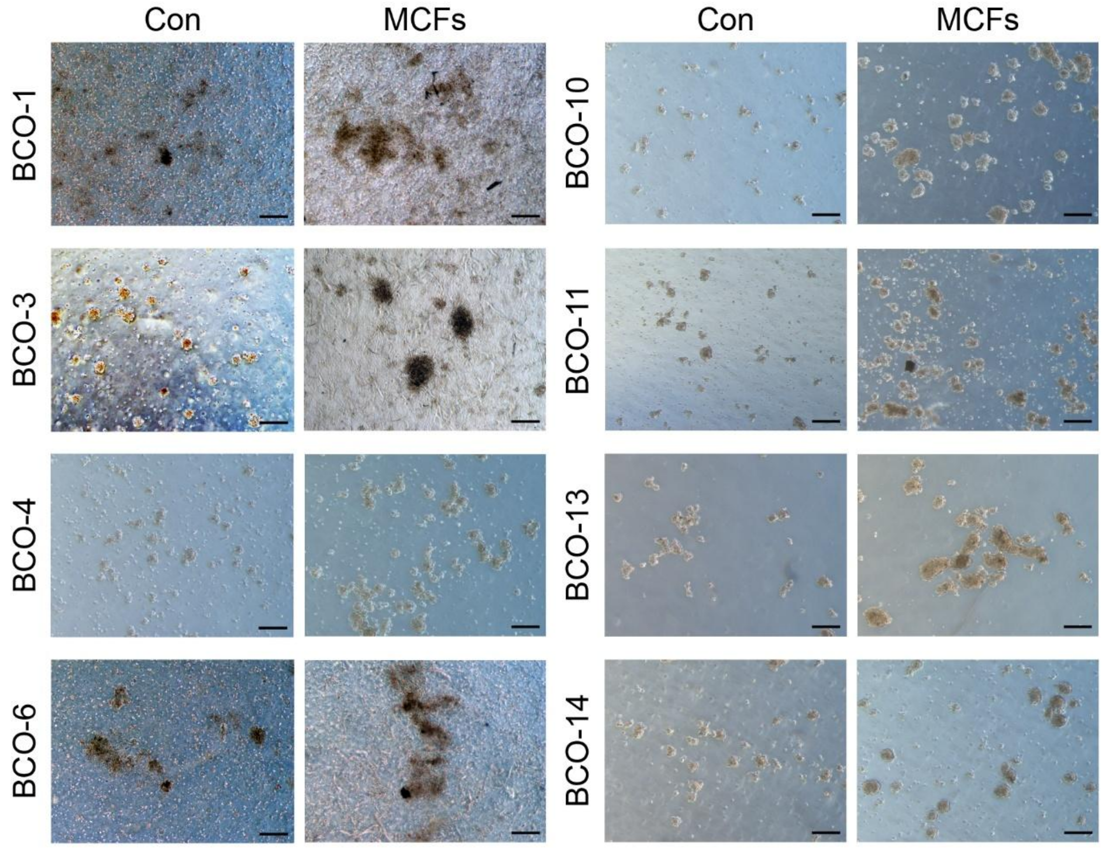

Figure S1. Enhanced viability and proliferation of BCOs with MCFs combined hydrogels in eight additional BCO lines, Related to Figure 2. Representative bright-field images of BCOs cultured for 10–14 days under control (mTG-crosslinked gelatin without MCFs) and MCFs conditions. Scale bar, 200 µm.

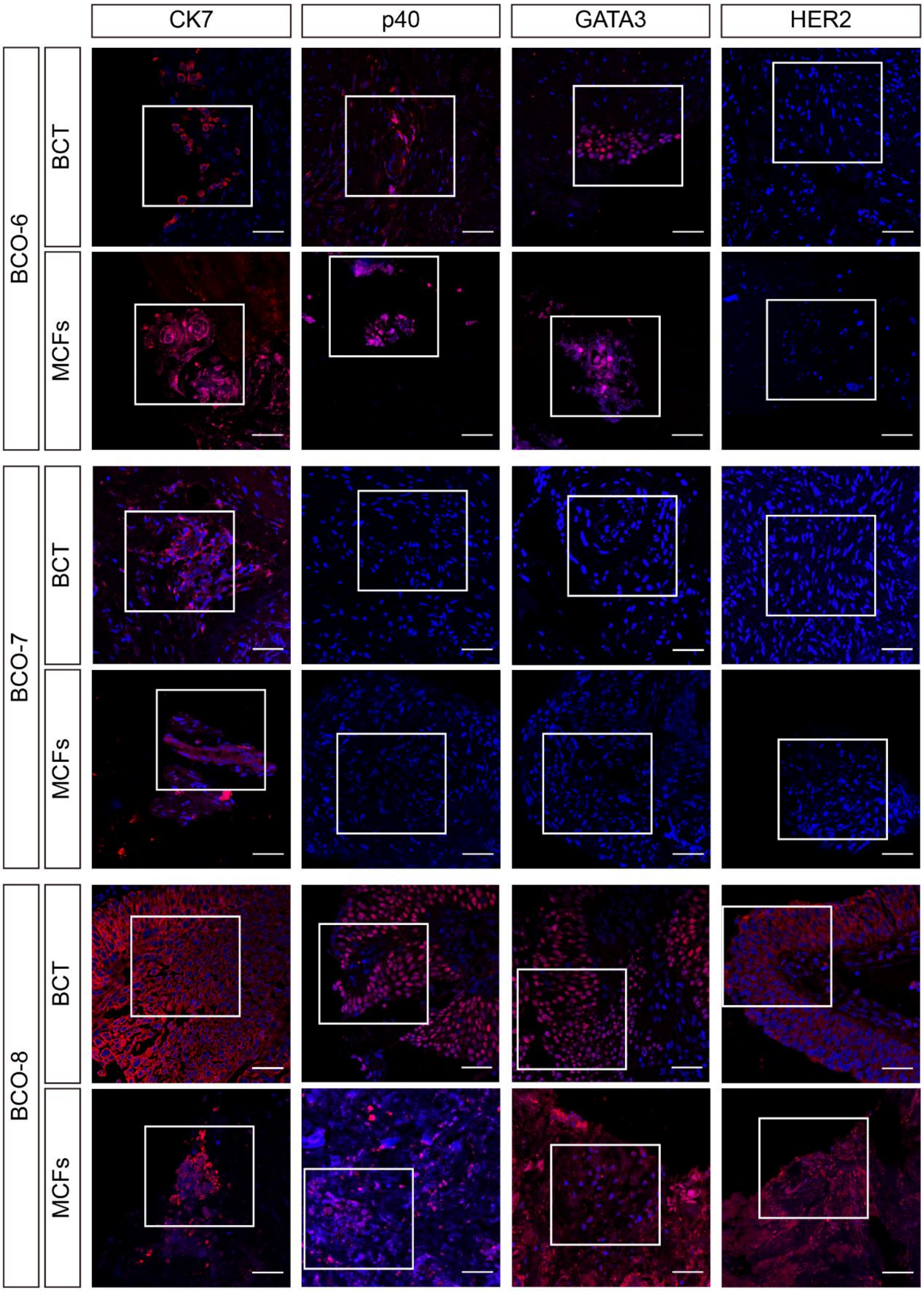

Figure S2. Representative immunofluorescence images of CK7, p40, GATA3, and HER2 in BCOs and corresponding BCTs with nuclear staining by DAPI (blue). The white boxes indicate regions corresponding to those shown in Figure 3B. Scale bar, 50 μm, Related to Figure 3.

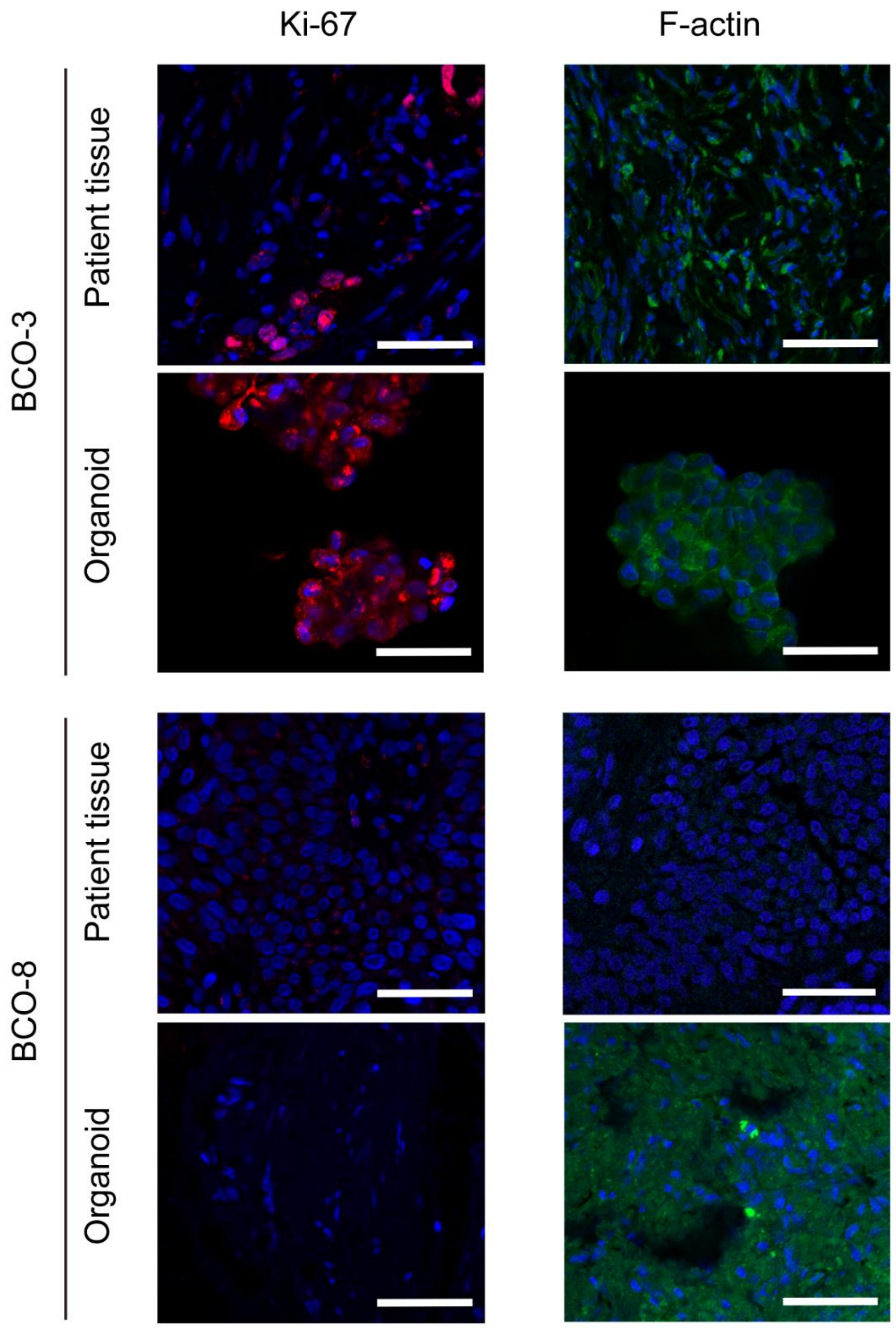

Figure S3. Representative immunofluorescence images of Ki-67 and F-actin in BCOs and corresponding parental tissues with nuclear staining by DAPI (blue) and F-actin staining (green); Scale bar, 50 μm, Related to Figure 3.

Supplemental Tables

Table S1. Composition of bladder cancer organoids medium, Related to Figure 2.

Reagent Name	Supplier	Working concentration
HEPES	Gibco	1×
Glutamax	Gibco	1×
penicillin/streptomycin	Beyotime	1×
B27	Gibco	1×
Primocin	Invitrogen	1×
N-acetyl-L-cysteine	MedChemExpress	1 mM
Wnt3a	GenScript	100 ng/mL
Recombinant Noggin protein	GenScript	0.1 μg/ml
Epidermal growth factor	GenScript	50 ng/ml
Fibroblast growth factor 10	GenScript	100 ng/ml
Nicotinamide	MedChemExpress	10 mM
SB202190	MedChemExpress	10 μM
A83-01	MedChemExpress	0.5 μM

The organoids medium is based on Advanced DMEM/F12 medium, supplemented with additional components as listed in the table

Table S2. Summary of clinical information from BCOs derived patients，Related to Figure 2.

ID	Stage	Age	Sex	Smoking status	Source	Pathological classification of parental tumor sample	Prior therapy
BCO-1	pT3N2	71	M	None	Robot-assisted radical cystectomy	High-grade, invasive	None
BCO-2	T3N0M0G3	57	F	None	Robot-assisted radical cystectomy	High-grade, invasive	Pirarubicin
BCO-3	T3N0M0G3	71	F	None	Laparoscopic partial cystectomy	High-grade, invasive	Pirarubicin
BCO-4	pT1N0M0	66	M	None	Laparoscopic partial cystectomy	High-grade papillary	None
BCO-5	pT1N0M0	76	F	None	Transurethral laser resection of the bladder	High-grade papillary	Adjuvant radiotherapy; Exemestane endocrine therapy
BCO-6	pT2aN0	70	M	None	Laparoscopic partial cystectomy	High-grade, invasive	None
BCO-7	T1N0M0	70	M	None	Robot-assisted radical cystectomy	High-grade, invasive	None
BCO-8	pTaN0M0	37	M	Former	Transurethral laser resection of the bladder	Low-grade papillary	None
BCO-9	T1N0M0G1	70	M	None	Transurethral Resection of Bladder Tumor	Low-grade papillary	None
BCO-10	T1N0M0	52	M	None	Transurethral Resection of Bladder Tumor	Low-grade urothelial, non-invasive	None
BCO-11	pT1G3	69	M	Now	Transurethral laser resection of the bladder	High-grade, invasive	R-CHOP; R-DHAP; BID+R-DICE
BCO-12	pT1N0M0	70	M	None	Transurethral Resection of Bladder Tumor	Low-grade papillary	None
BCO-13	pT1G3N0M0	74	F	None	Transurethral Resection of Bladder Tumor	high-grade papillary	None
BCO-14	pTaN0M0	76	F	None	Transurethral laser resection of the bladder	Low-grade	None

M: male, F: female

Table S3. Summary of passage information for experimental samples, Related to Figure 2, Figure 3, Figure 4, Figure 5, Figure 6, and Figure 7.

ID	Growth experiment	Migration and invasion experiment	HE staining and IF	WES	Clinically relevant drug test	Postoperative bladder drug instillation experiment	Long-term and short-term growth experiment	Long-term and short-term HE staining	Long-term and short-term culture in clinically relevant drug test
BCO-1	2	–	–	2	–	–	–	–	–
BCO-2	–	–	–	2	–	–	–	–	–
BCO-3	2	2	–	2	–	2	Early: 2; Later: 8	Early: 2; Later: 8	Early: 2; Later: 8
BCO-4	2	2	2	–	2	–	–	–	–
BCO-5	–	2	–	–	–	2	–	–	–
BCO-6	2	–	2	–	–	–	–	–	-
BCO-7	–	–	2	2	–	–	Early: 2; Later: 7	Early: 2; Later: 7	Early: 2; Later: 7
BCO-8	2	–	2	2	2	–	–	–	–
BCO-9	2	–	–	–	–	2	–	–	–
BCO-10	2	–	–	–	–	–	Early: 2; Later: 8	Early: 2; Later: 8	Early: 2; Later: 8
BCO-11	2	–	–	–	–	2	–	–	–
BCO-12	2	–	–	2	2	–	–	–	–
BCO-13	2	–	–	–	–	–	–	–	–
BCO-14	2	–	–	–	–	–	–	–	–

Table S4. Postoperative irrigation status and cystoscopy findings of patients corresponding to BCOs, Related to Figure 5.

ID	Postoperative irrigation	Time of performing cystoscopy after surgery	Result of cystoscopy
BCO-3	Pirarubicin	3 months 19 days	Normal
BCO-5	Pirarubicin	1 months 11 days	Normal
BCO-9	Mitomycin C	3 months 7 days	Normal
BCO-11	Pirarubicin	4 months	Normal
